# E3 ubiquitin ligase TRIM21-mediated K48-linked ubiquitination of ALDH2 rs671 mutant promotes adverse cardiac remodeling

**DOI:** 10.1172/jci.insight.197555

**Published:** 2026-02-24

**Authors:** Tianrui Han, Xin Wen, Yunyun Guo, Xiangkai Zhao, Jian Zhang, Yuguo Chen, Feng Xu

**Affiliations:** 1 Department of Emergency Medicine, Qilu Hospital of Shandong University, Jinan, China.; 2 Shandong Provincial Clinical Research Center for Emergency and Critical Care Medicine, Institute of Emergency and Critical Care Medicine of Shandong University, Chest Pain Center, Qilu Hospital of Shandong University, Jinan, China.; 3 Medical and Pharmaceutical Basic Research Innovation Center of Emergency and Critical Care Medicine, China’s Ministry of Education, Shandong Provincial Engineering Laboratory for Emergency and Critical Care Medicine, Key Laboratory of Emergency and Critical Care Medicine of Shandong Province, Key Laboratory of Cardiopulmonary-Cerebral Resuscitation Research of Shandong Province, Qilu Hospital of Shandong University, Jinan, China.; 4 NMPA Key Laboratory for Clinical Research and Evaluation of Innovative Drug, Qilu Hospital of Shandong University, Jinan, China.; 5 National Key Laboratory for Innovation and Transformation of Luobing Theory, The Key Laboratory of Cardiovascular Remodeling and Function Research, Chinese Ministry of Education, Chinese National Health Commission and Chinese Academy of Medical Sciences, Qilu Hospital of Shandong University, Jinan, China.

**Keywords:** Cardiology, Cell biology, Fibrosis, Heart failure, Ubiquitin-proteosome system

## Abstract

Heart failure (HF) persists as the primary cause of death among patients recovering from acute myocardial infarction (AMI). Protein ubiquitination has been implicated as a key modulator of HF pathogenesis, yet the role of ubiquitination in the *Aldh2* rs671 mutant — the most common single-nucleotide variant in human populations — remains poorly understood. We discovered TRIM21 as a previously unrecognized E3 ubiquitin ligase for the ALDH2 rs671 mutant and elucidated its mechanistic involvement in HF progression. Using *Aldh2* BM chimeric mice to model AMI, we observed that WT mice transplanted with *Aldh2* rs671 donor BM developed severe myocardial fibrosis and markedly reduced cardiac systolic function 2 weeks after infarction compared with controls. This phenotype arose from defective macrophage efferocytosis caused by myeloid-specific *Aldh2* rs671 mutation. Through high-resolution mass spectrometry proteomics, we identified TRIM21 as the E3 ligase targeting ALDH2. TRIM21 catalyzed K48-linked ubiquitination at ALDH2 lysine 73. Macrophage-specific *Trim21* knockdown via AAV-sh*Trim21* reversed both the exacerbated cardiac fibrosis and systolic dysfunction by restoring macrophage efferocytosis. These findings delineate the upstream E3 ubiquitin ligase and the ubiquitination site of ALDH2, revealing a potential therapeutic target for HF.

## Abstract 2

TRIM21-mediated increased ubiquitination of ALDH2 rs671 protein results in post-MI adversed cardiac remodeling via suppressing macrophages efferocytosis.

## Introduction

Heart failure (HF) is the primary cause of mortality among patients with acute myocardial infarction (MI) (1, 2). After MI, despite the availability of interventions such as primary percutaneous coronary intervention, i.v. thrombolysis, and other reperfusion strategies that effectively restore myocardial blood supply, post-MI HF remains associated with high mortality and disability rates (3). The pathological progression after MI can be delineated into 3 phases: acute phase (≤ 7 days), subacute phase (> 7 days and < 28 days), and chronic phase (≥ 28 days). Firstly, pressure and volume overload elevate ventricular wall stress and impair left ventricular contractility within 12 hours. Subsequently, inflammatory cells infiltrate the damaged tissue between days 1 and 3 to clear necrotic cells and trigger repair response. This initial proinflammatory phase transitions into an antiinflammatory and reparative stage from days 3 to 7, promoting tissue healing and scar formation, which prevents cardiac rupture. Progressive cardiomyocyte loss and interstitial fibrosis occur at weeks 1 to 3. Finally, fibrous scar tissue matures at 1 month, followed by sustained chronic inflammation, accomplishing the transition from myocardium infarction to adverse remodeling (4–6). Although adaptive cardiac remodeling is essential for reducing early mortality after MI, excessive and maladaptive ventricular remodeling negatively affects ventricular size and function, culminating in the clinical syndrome of HF (7).

Following MI, monocyte and macrophage populations at the infarct site generally return to baseline levels after 14 days, but these cells may persist in the remote myocardium for several months (8, 9). Monocytes and macrophages are integral in both the initial inflammatory response to injury and subsequent wound healing in various tissues, including the heart. Nevertheless, dysfunctional immune cell activity was implicated in adverse ventricular remodeling in mouse models, prompting ongoing research into immunomodulatory strategies aimed at averting the progression to HF (7).

Previous clinical studies have demonstrated that the *Aldh2* rs671 variant is associated with an elevated risk of major adverse cardiac events (MACE), including HF (10). In parallel, accumulating experimental evidence indicates that enhanced ALDH2 activity protects against MI through multiple mechanisms, such as attenuation of ischemia/reperfusion injury, detoxification of reactive aldehydes, modulation of oxidative stress, and suppression of cardiac fibrosis (11, 12). However, the precise mechanisms by which the ALDH2 rs671 variant affects post-MI cardiac remodeling remain incompletely understood. Furthermore, in consideration of several different pathological processes after MI, our study conducted a time-course animal study to discover the most potent periods during which *Aldh2* rs671 variant shows effects on MI.

## Results

### Efferocytosis-derived cardiac fibrosis was enhanced by myeloid-specific Aldh2 rs671 mutation.

Initially, a mouse MI model was established and assessed by cardiac ultrasound and histologic morphology at different time points. The probability of survival was decreased in *Aldh2* rs671 mice (Supplemental Figure 1A; supplemental material available online with this article; https://doi.org/10.1172/jci.insight.197555DS1). Furthermore, worse cardiac function and increased cardiac fibrosis were observed in *Aldh2* rs671 mice compared with WT mice and, more distinctly, at 14 days after LAD ligation (Supplemental Figure 1, B–E). These results indicate that the *Aldh2* rs671 variant had a greater effect on cardiac fibrosis after MI.

**Figure 1:**
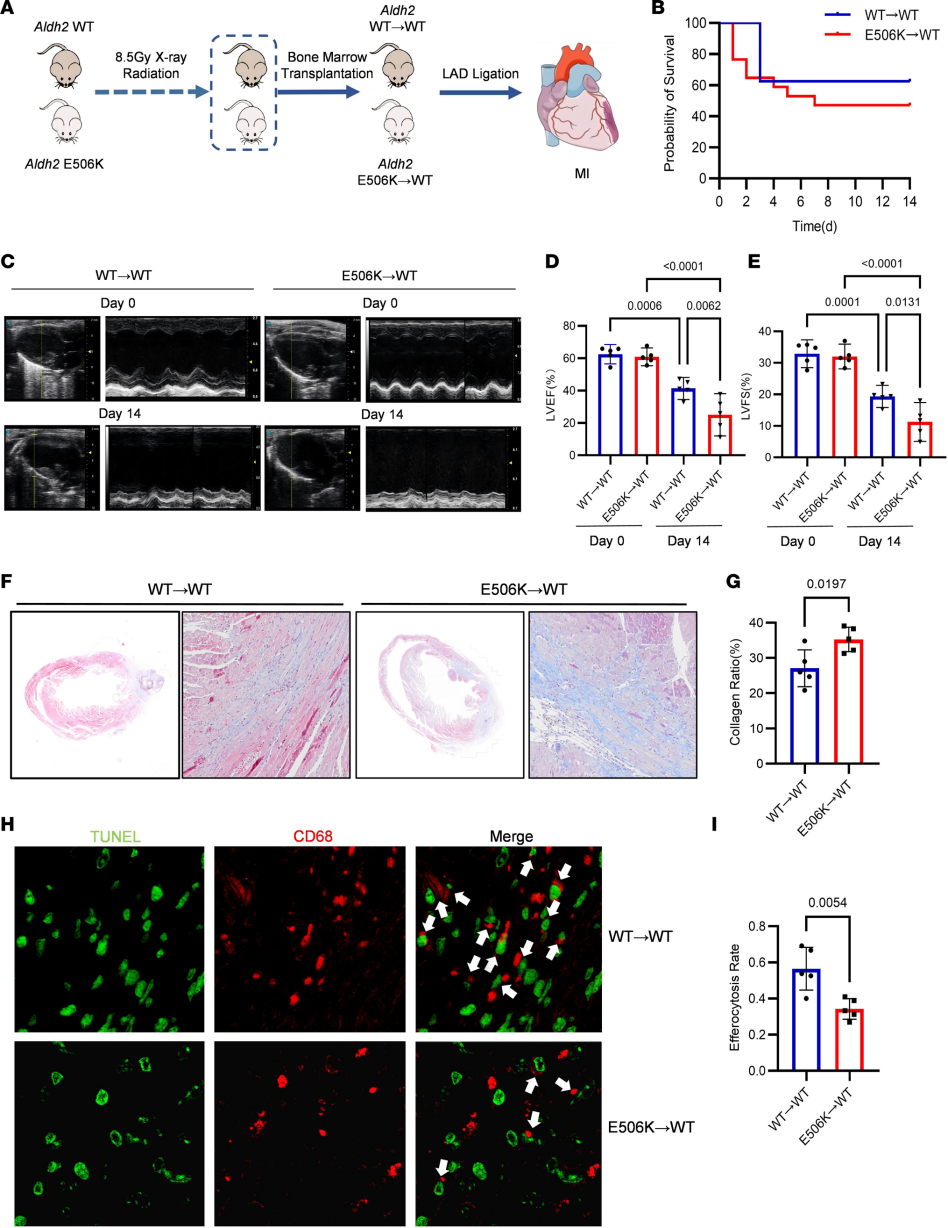
Post-MI cardiac fibrosis is enhanced by myeloid-specific *Aldh2* rs671 variant. (**A**) Experimental strategy of myeloid-specific *Aldh2* rs671 mice construction. (**B**) Probability of survival curve of WT mice transplanted WT or rs671 mice BM after MI operation. (**C**–**E**) Representative parasternal long-axis views and M-mode images. Echocardiographic analysis of ejection fraction (EF) and fractional shortening (FS) on days 0 and 14 after MI or sham operation in WT mice transplanted WT or rs671 mice BM (*n* = 5). (**F** and **G**) Representative Masson trichrome staining of cardiac tissue obtained from WT mice transplanted WT or rs671 mice BM on day 14 after MI operation. Quantitative analysis of collagen ratio on day 14 after MI operation (*n* = 5) (**F**) Scale bar 100 μm. (**G**) Scale bar 20μm. (**H** and **I**) Representative photomicrographs of terminal deoxynucleotidyl transferase dUTP nick-end labeling (TUNEL, green) staining with macrophages marker CD68 (red) in cardiac tissue obtained from WT and rs671 mice on day 14 after MI operation. Analysis of internalization of apoptotic cardiomyocytes in macrophages. Macrophages that locate close to apoptotic cells are scored as having internalized apoptotic cardiomyocytes (*n* = 5). White arrows indicate efferocytotic macrophages. Data are expressed as mean ± SEM. One-way ANOVA and Tukey post hoc test were used for analysis.

To determine whether macrophages contribute to in the enhanced cardiac fibrosis associated with the *Aldh2* rs671 variant, BM derived from WT or rs671 mice was transplanted into irradiated WT recipients. MI was induced and cardiac function, histological morphology, and survival were assessed (Figure 1, A and B). Mice receiving the rs671 BM exhibited impaired cardiac systolic function and more severe cardiac fibrosis compared with those receiving the WT BM (Figure 1, C–G). As shown in Supplemental Figure 1, total ALDH2 rs671 mutation reduced cardiac systolic function by about 43%, while myeloid-specific rs671 mutation resulted in about 39% reduction. The extent of phenotype change resulting from total rs671 mutation and myeloid-specific rs671 mutation is approximately the same. Furthermore, immunofluorescence staining combined with TUNEL analysis showed efferocytosis deficiency in ischemic heart tissue from mice receiving the rs671 BM (Figure 1, H and I). Similar findings were observed in ischemic heart tissue and BM-derived macrophages (BMDMs) between WT and rs671 mice indicating that impaired efferocytosis contributes to cardiac fibrosis, while increased cardiomyocyte apoptosis was also observed in rs671 mice (Supplemental Figure 2, A–F). Accordingly, we focused on macrophages function in this study. In addition, we marked resident cardiac macrophages by TIM-4 and total macrophages by MOMA-2 to quantify resident macrophages and infiltrating macrophages from mice receiving ALDH2 rs671 or WT BM by immunofluorescence. The result showed no marked differences in the quantities of resident or infiltrating macrophages between mice receiving ALDH2 rs671 or WT BM following transplantation (Supplemental Figure 2, G and H).

### Efferocytosis deficiency was related to increased degradation of ALDH2 rs671 protein.

**Figure 2:**
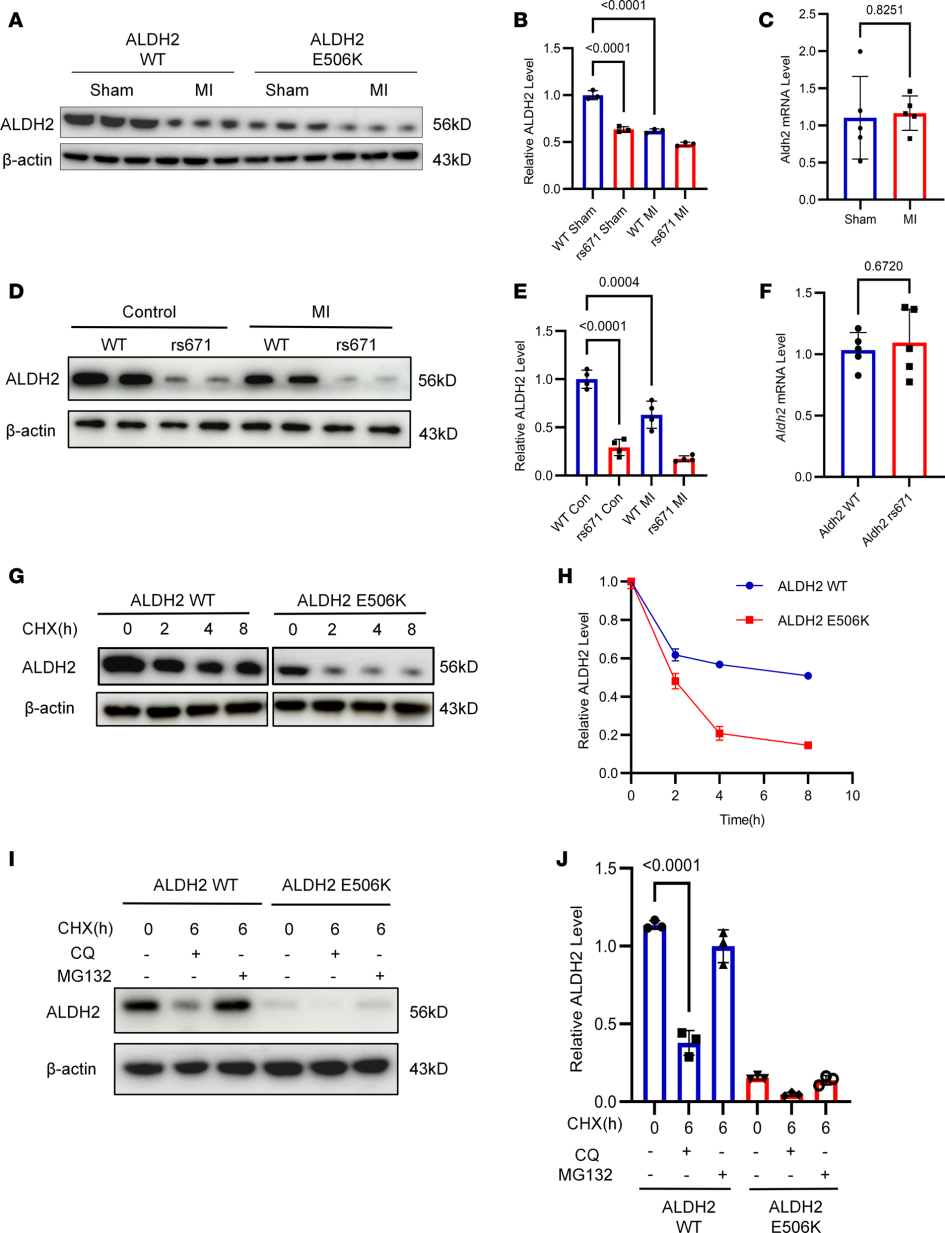
Degradation of ALDH2 protein undergoes proteosomal pathway. (**A** and **B**) Representative bands and quantification of ALDH2 protein level, obtained from ischemic and nonischemic heart tissue from WT and rs671 mice (*n* = 3). (**C**) Quantitative analysis of relative *Aldh2* mRNA expression in ischemic heart tissue obtained from WT and rs671 mice (*n* = 5). (**D** and **E**) Representative bands and quantification of ALDH2 protein level was obtained from PBMCs of *Aldh2* WT and rs671 individuals with or without MI (*n* = 4). (**F**) Quantitative analysis of relative *Aldh2* mRNA expression in peripheral blood mononuclear cells (PBMCs) obtained from *Aldh2* WT and rs671 patients with MI (*n* = 5). (**G**–**J**) BMDMs obtained from WT and rs671 mice were treated with cycloheximide (CHX) for the indicated times, some of which was followed by MG132 or chloroquine (CQ) stimulation (*n* = 3). β-Actin was used as loading control. Data are expressed as mean ± SEM. Unpaired 2-tailed Student’s *t* test was used for the analysis in **C** and **F**. Two-way ANOVA and Tukey post hoc test were used for the analysis in **H**. One-way ANOVA and Tukey post hoc test were used for the analysis in **B**, **E**, and **J**.

Our previous study has shown that the *Aldh2* rs671 variant inhibits efferocytosis during atherosclerosis by enhancing the degradation of Rac2 protein (13). Immunofluorescence showed that the same regulatory process also occurred after MI (Supplemental Figure 3, A and B). Moreover, upregulated ubiquitination and increased degradation of Rac2 were observed after MI, with rs671 protein showed even more ubiquitin level and less expression compared with WT protein (Supplemental Figure 3C). However, we observed that expression of ALDH2 rs671 mutant decreased obviously compared with WT protein. Therefore, we detected the ALDH2 protein level in both ischemic and nonischemic heart tissue from WT and rs671 mice. Compared with normal heart tissue, a remarkable decrease in ALDH2 protein level was discovered in ischemic heart tissue (Figure 2, A and B). To exclude the possibility that *Aldh2* gene shows different expression levels at the mRNA stage, we also performed qPCR in mice heart tissue and no marked change was observed between WT and rs671 variant (Figure 2C). Therefore, we examined the expression level of ALDH2 protein obtained from peripheral blood mononuclear cells (PBMCs) derived from patients with MI and patients with unstable angina. We affirmed that ALDH2 rs671 protein exhibited less expression than the WT protein, while MI further dampened expression of ALDH2 protein (Figure 2, D and E). We also performed qPCR in human PBMCs and found no change between WT and rs671 variant in *Aldh2* mRNA level (Figure 2F). In summary, the decreased ALDH2 protein level was due to degradation.

Initially, we treated BMDMs with the de novo protein synthesis inhibitor cycloheximide and measured the degradation of ALDH2. The degradation of ALDH2 was obviously increased in rs671 macrophages compared with that in WT macrophages (Figure 2, G and H). To verify the degradation pathway of the ALDH2 protein, we treated BMDMs with chloroquine or MG132 to inhibit the lysosomal or proteasomal degradation pathways. Results show that MG132 treatment substantially reduced the degradation of ALDH2, while chloroquine had no effect on ALDH2 degradation (Figure 2, I and J). These results reveal that ALDH2 mainly underwent proteasomal degradation.

### Ubiquitin E3 ligase TRIM21 regulated ALDH2 protein ubiquitination.

**Figure 3:**
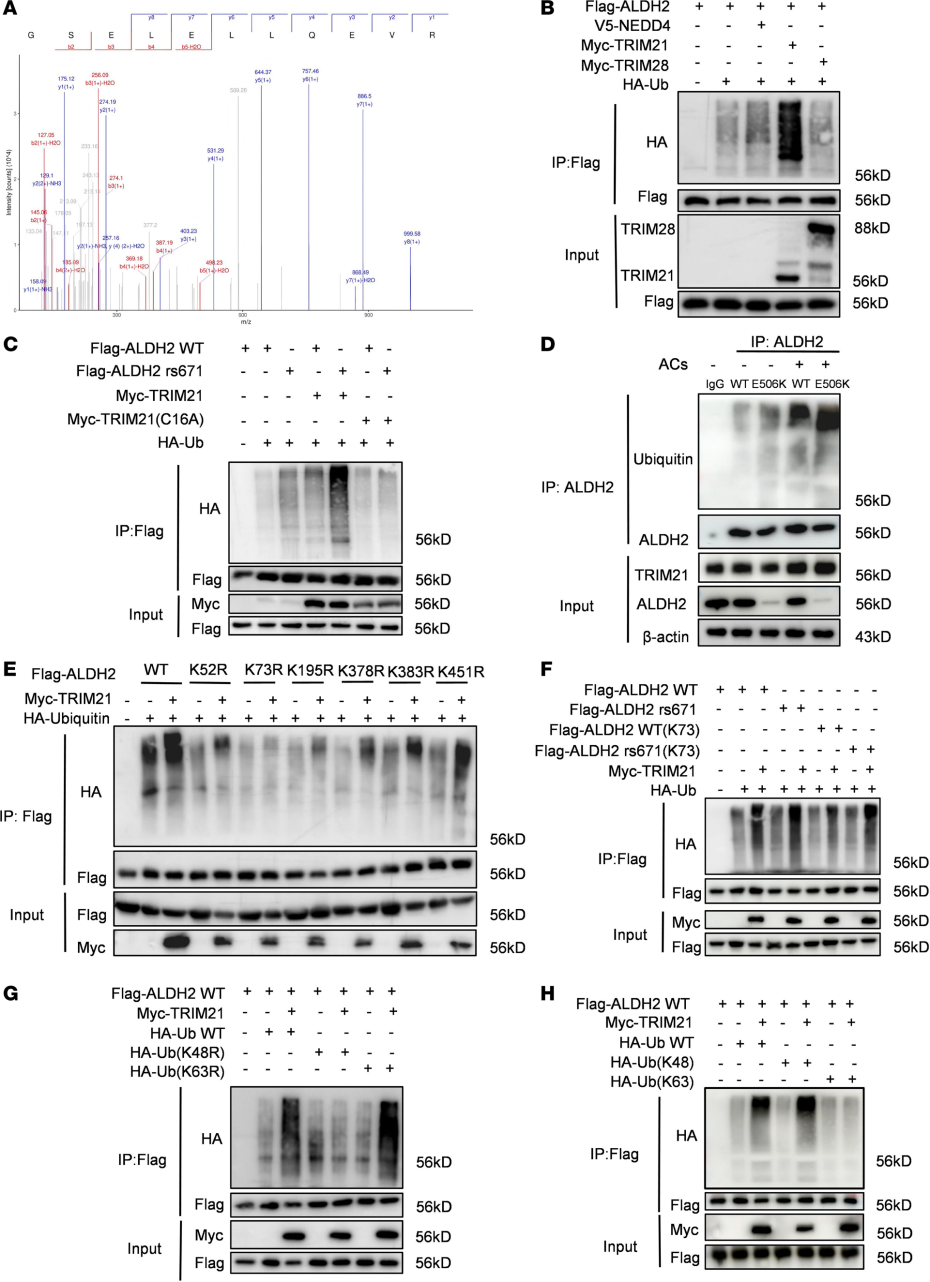
Ubiquitination of ALDH2 protein is mediated by E3 ligase TRIM21. (**A**) TRIM21 was identified in the protein mixture enriched by ALDH2 antibody. Graph represented peptide fragment GSELELLQEVR. (**B**) V5-NEDD4, Myc-TRIM21, and Myc-TRIM28 were separately cotransfected into HEK293T cells with Flag-ALDH2 and HA-ubiquitin. Cell lysates were subjected to coimmunoprecipitation (Co-IP) with anti-Flag antibody and followed by Western blotting. (**C**) Myc-TRIM21 and Myc-C16A were cotransfected into HEK293T cells with Flag-ALDH2, Flag-rs671, and HA-ubiquitin. Cell lysates were subjected to Co-IP with anti-Flag antibody and followed by Western blotting. (**D**) Co-IP using anti-ALDH2 antibody was performed with lysates from *Aldh2* rs671 or WT mice BMDMs treated with or without apoptotic cells (ACs) followed by Western blotting. β-Actin were used as loading controls. (**E**) HEK293T cells were transfected with HA-Ub and Flag-ALDH2 or its single site mutation, with or without Myc-TRIM21. Cell lysates were subjected to Co-IP with anti-Flag antibody and followed by Western blotting. (**F**) Flag-ALDH2, Flag-rs671, Flag-ALDH2 (K73), and Flag-rs671 (K73) were cotransfected into HEK293T cells with Myc-TRIM21 and HA-ubiquitin. Cell lysates were subjected to Co-IP with anti-Flag antibody and followed by Western blotting. (**G**) HA-ubiquitin, HA-ubiquitin (K48R), and HA-ubiquitin (K63R) were cotransfected into HEK293T cells with Flag-ALDH2 and Myc-TRIM21. Cell lysates were subjected to Co-IP with anti-Flag antibody and followed by Western blotting. (**H**) HA-ubiquitin, HA-ubiquitin (K48) and HA-ubiquitin (K63) were cotransfected into HEK293T cells with Flag-ALDH2 and Myc-TRIM21. Cell lysates were subjected to Co-IP with anti-Flag antibody and followed by Western blotting.

To discover the probable upstream ubiquitination pathway of ALDH2, we performed high-resolution mass spectrometry–based proteomics using primary macrophages treated with apoptotic cells (Figure 3A). The results identified 3 possible molecules interacting with ALDH2. We further cotransfected HEK293T cells with Flag-ALDH2 and HA-ubiquitin along with Myc-TRIM21, Myc-TRIM28, or V5-NEDD4. Among these, only TRIM21 was found to regulate the ubiquitination of ALDH2 (Figure 3B).

Moreover, we used a Myc-TRIM21 C16A mutant plasmid to confirm the regulatory effect of TRIM21 on ALDH2 rs671 or WT protein, discovering a higher ubiquitination level in rs671 protein compared with WT protein (Figure 3C). To corroborate these findings in a physiologically relevant context, we utilized BMDMs derived from WT and rs671 mice treated with or without apoptotic cells to perform coimmunoprecipitation (Co-IP) between ALDH2 and ubiquitin, confirming that the ubiquitination level of ALDH2 increased during efferocytosis (Figure 3D). To verify which ubiquitination site of ALDH2 TRIM21 modulated, we constructed K52R, K73R, K195R, K378R, K383R, and K451R of ALDH2 and cotransfected with or without HA-ubiquitin and Myc-TRIM21. Results showed that K73R was resistant to TRIM21 modulating ubiquitination (Figure 3E). Furthermore, we constructed K73 mutation plasmids of both ALDH2 WT and rs671 and cotransfected with or without HA-ubiquitin and Myc-TRIM21 again. Results indicate that K73 mutation did not block ubiquitination of ALDH2, and ALDH2 rs671 K73 mutant still exhibited higher ubiquitination level than WT K73 mutant (Figure 3F). These results imply that ALDH2 mainly modulated the ubiquitination of ALDH2 at lysine 73.

To investigate the type of TRIM21-mediated ubiquitination of ALDH2, we used vectors expressing HA-tagged ubiquitin K48, K63, K48R, and K63R mutant to be cotransfected with Flag-ALDH2, and with or without Myc-TRIM21. Results showed that TRIM21 increased the ubiquitination of ALDH2 with the K63R-ubiquitin plasmid rather than the K48R-ubiquitin plasmid, while ALDH2 cotransfected with K48-ubiquitin showed raised ubiquitination level compared with ALDH2 cotransfected with K63-ubiquitin (Figure 3, G and H). Through results above, we confirmed that TRIM21 modulates K48-linked polyubiquitination of ALDH2. Additionally, we reconfirmed that ALDH2 also regulated K48-link polyubiquitination of Rac2 (Supplemental Figure 3D).

**Figure 4:**
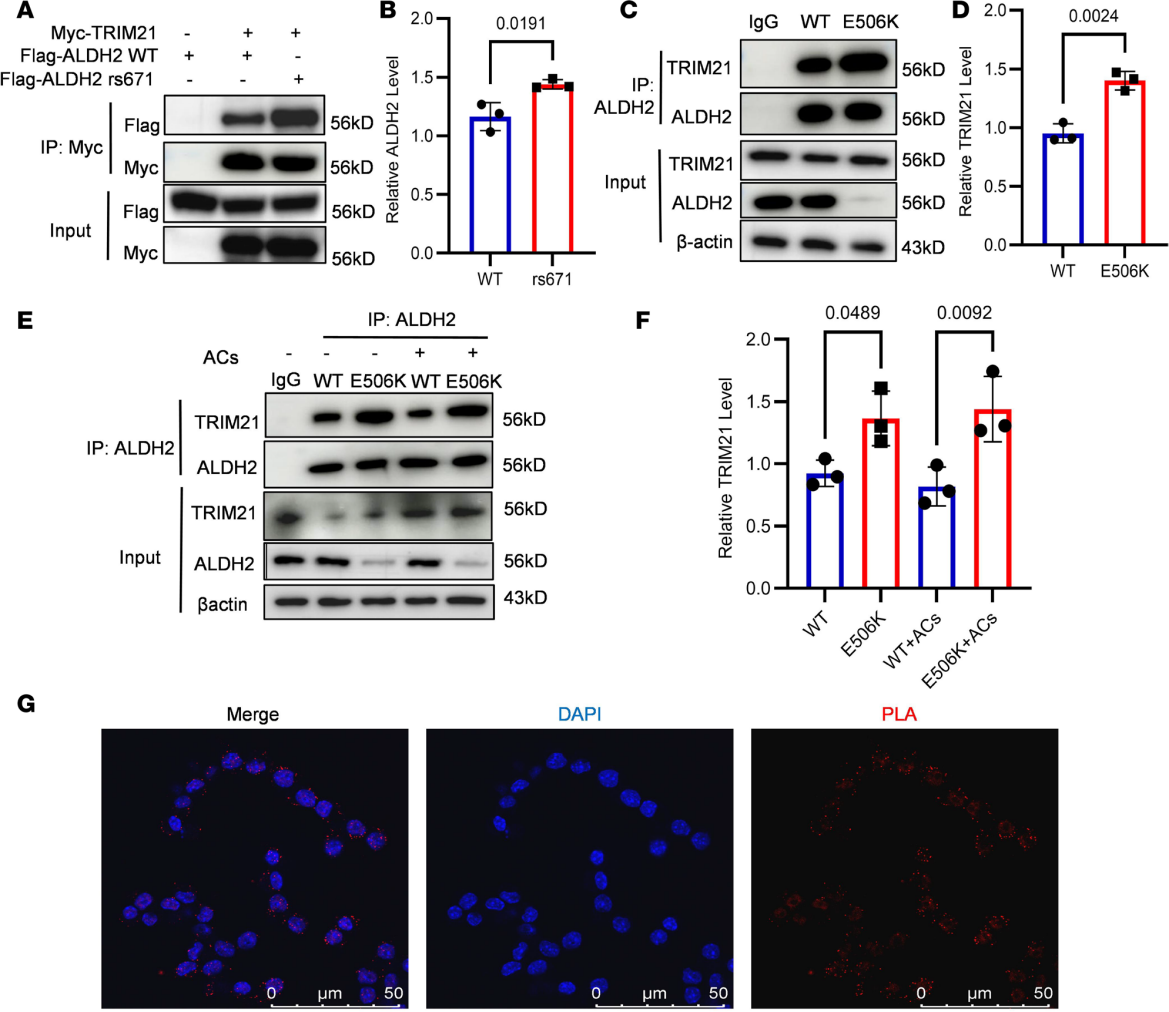
TRIM21 directly interacts with ALDH2. (**A** and **B**) Flag-ALDH2 or Flag-rs671 were cotransfected into HEK293T cells with Myc-Rac2. Cell lysates were subjected to Co-IP with anti-Myc antibody and followed by Western blotting. Quantitative analysis of relative ALDH2 level of lysates (*n* = 3). (**C** and **D**) Co-IP using anti-ALDH2 antibody was performed with lysates from WT and rs671 mice BMDMs. Quantitative analysis of relative TRIM21 level of lysates (*n* = 3). (**E** and **F**) Co-IP using anti-ALDH2 antibody was performed with lysates from WT and rs671 mice BMDMs treated with or without apoptotic cells. Quantitative analysis of relative TRIM21 level of lysates (*n* = 3). (**G**) Proximity ligation assay (PLA) of TRIM21 and ALDH2 in mice primary peritoneal macrophages. Binding TRIM21 and ALDH2 are red-stained. Scale bar 50 μm. β-Actin were used as loading controls. Data are expressed as mean ± SEM. Unpaired 2-tailed Student’s *t* test were used for the analysis in **B** and **D**. One-way ANOVA and Tukey post hoc test were used for the analysis in **F**.

To detect whether TRIM21 interacts directly with ALDH2, we transfected Flag-ALDH2 or Flag-rs671 with Myc-TRIM21 into HEK293T cells. In Co-IP assays, we observed that TRIM21 interacted with ALDH2, and increased binding was found between the rs671 mutant and TRIM21 (Figure 4, A and B). We also found that TRIM21 expression does not differ between WT mice and those carrying the ALDH2 rs671 mutant, which is validated in human PBMCs (Supplemental Figure 4A). These results indicate that higher ubiquitination of ALDH2 rs671 mutant is due to increased binding between the rs671 mutant and TRIM21. In vivo Co-IP was also performed to confirm the results above (Figure 4, C and D). We further assessed the direct interaction between TRIM21 and ALDH2 in BMDMs treated with apoptotic cells, finding no remarkable change of binding ability between TRIM21 and ALDH2 during the process of efferocytosis (Figure 4, E and F). However, we observed that apoptotic cell treatment slightly increased TRIM21 expression, which is consistent with Figure 3D. Conclusively, increased ubiquitination level of ALDH2 caused by apoptotic cells treatment is mainly due to higher TRIM21 expression level but not increasing binding with TRIM21 and ALDH2. Finally, in order to verify the direct interaction between TRIM21 and ALDH2, we did a proximity ligation assay (PLA) between the 2 proteins and then used confocal microscopy to capture the images for analysis. Result show that TRIM21 did have direct interaction with ALDH2 (Figure 4G).

### Macrophage-specific Trim21 downregulation rescued deficient efferocytosis-derived cardiac fibrosis owing to Aldh2 rs671 variant.

**Figure 5:**
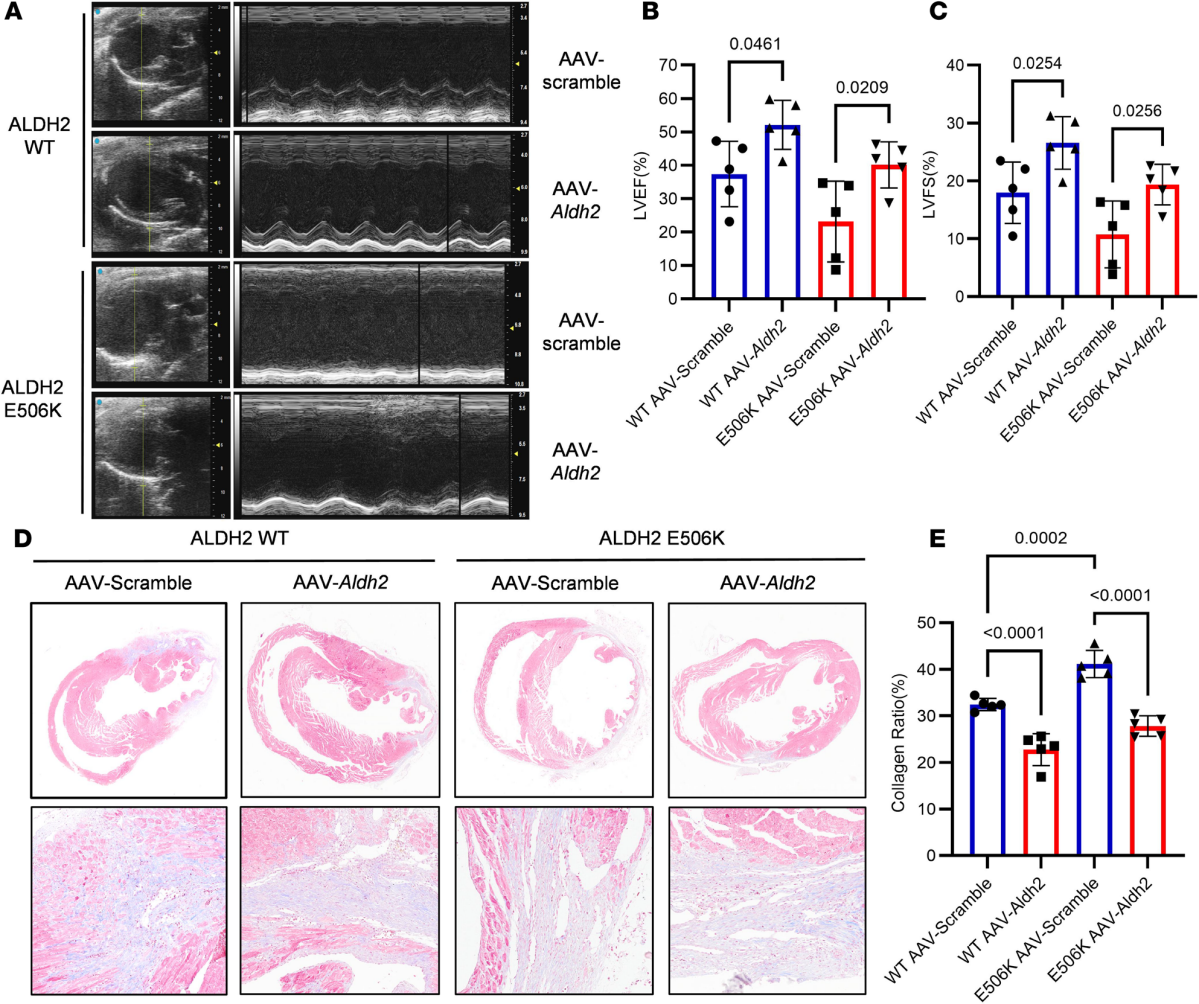
*Aldh2* overexpressing rescues worsened cardiac function induced by *Aldh2* rs671 variant. (**A**–**C**) Representative parasternal long-axis views and M-mode images. Echocardiographic analysis of LVEF and LVFS on days 14 after MI in WT and rs671 mice injected with AAV-scramble or AAV-*Aldh2* (*n* = 5). (**D** and **E**) Representative Masson trichrome staining of cardiac tissue obtained from WT and rs671 mice injected with AAV-scramble or AAV-*Aldh2* on day 14 after MI operation. Quantitative analysis of collagen ratio on day 14 after MI operation (*n* = 5). Data are expressed as mean ± SEM. One-way ANOVA and Tukey post hoc test were used for analysis. (**D**) Scale bar 100 μm.

After clarifying the interaction between TRIM21 and ALDH2, we used AAV-*Aldh2* to carry out rescue experiments. AAV-scramble and AAV-*Aldh2* were injected into 6-week-old WT and rs671 mice via the tail vein, and an MI model was established 2 weeks after virus injection. Infection rate of AAV was detected by examining ALDH2 expression level in BMDMs obtained from injected mice (Supplemental Figure 4B). We found that overexpression of ALDH2 was able to relieve the descending range of cardiac function after MI and mitigated cardiac fibrosis during cardiac repair (Figure 5, A–E). Efferocytosis rate was also enhanced by overexpression of ALDH2 after MI (Supplemental Figure 5, A and B).

**Figure 6:**
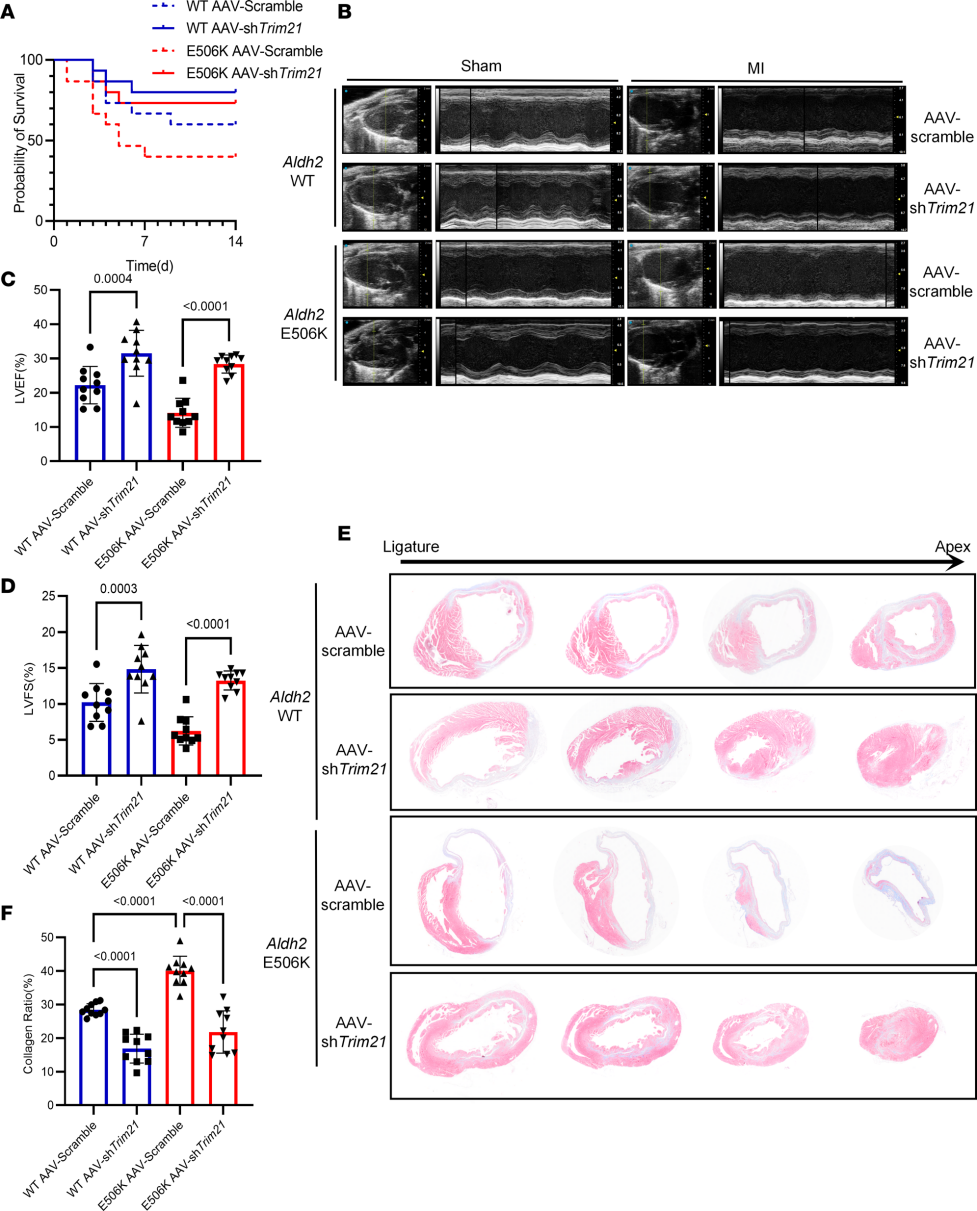
Macrophage-specific *Trim21* downregulating ameliorates cardiac fibrosis owing to *Aldh2* rs671 variant. (**A**) Probability of survival curve of *Aldh2* WT and rs671 mice injected with CD68-promoter AAV-scramble or AAV-sh*Trim21* after MI operation. (**B**–**D**) Representative parasternal long-axis views and M-mode images. Echocardiographic analysis of LVEF and LVFS on days 14 after MI or sham operation in WT and rs671 mice injected with CD68-promoter AAV-scramble or AAV-sh*Trim21* (*n* = 10). (**E** and **F**) Representative Masson trichrome staining of cardiac tissue obtained from WT and rs671 mice injected with CD68-promoter AAV-scramble or AAV-sh*Trim21* on days 14 after MI operation. Quantitative analysis of collagen ratio on day 14 after MI operation (*n* = 10). Data are expressed as mean ± SEM. One-way ANOVA and Tukey post hoc test were used for analysis.

To further elucidate the role of TRIM21 in macrophages during cardiac fibrosis following MI, we generated *Trim21* macrophage–specific silencing mice using adeno-associated virus (AAV). AAV-scramble and AAV-sh*Trim21* using the CD68 promoter were injected into 6-week-old WT and rs671 mice via the tail vein, and an MI model was established 2 weeks after virus injection. The findings indicate that the TRIM21 protein level decreased significantly after AAV-sh*Trim21* injection (Supplemental Figure 4C). First, primary peritoneal macrophages from these mice were treated with apoptotic cells. Immunofluorescence showed that downregulating *Trim21* rescued the deficient efferocytosis induced by the *Aldh2* rs671 variant (Supplemental Figure 4, D and E). We also observed the probability of survival of these mice (Figure 6A).

Furthermore, we assessed cardiac function and histological morphology. Compared with mice injected with AAV-scramble, mice injected with AAV-sh*Trim21* displayed mitigated cardiac function (Figure 6, B–D). In addition, cardiac fibrosis was alleviated after downregulating *Trim21* (Figure 6, E and F). We also observed increased efferocytosis after *Trim21* downregulation by immunofluorescence (Supplemental Figure 5, C and D). These results reveal that the rs671 mutant-mediated cardiac fibrosis deficiency was improved by downregulating *Trim21*.

## Discussion

**Figure 7:**
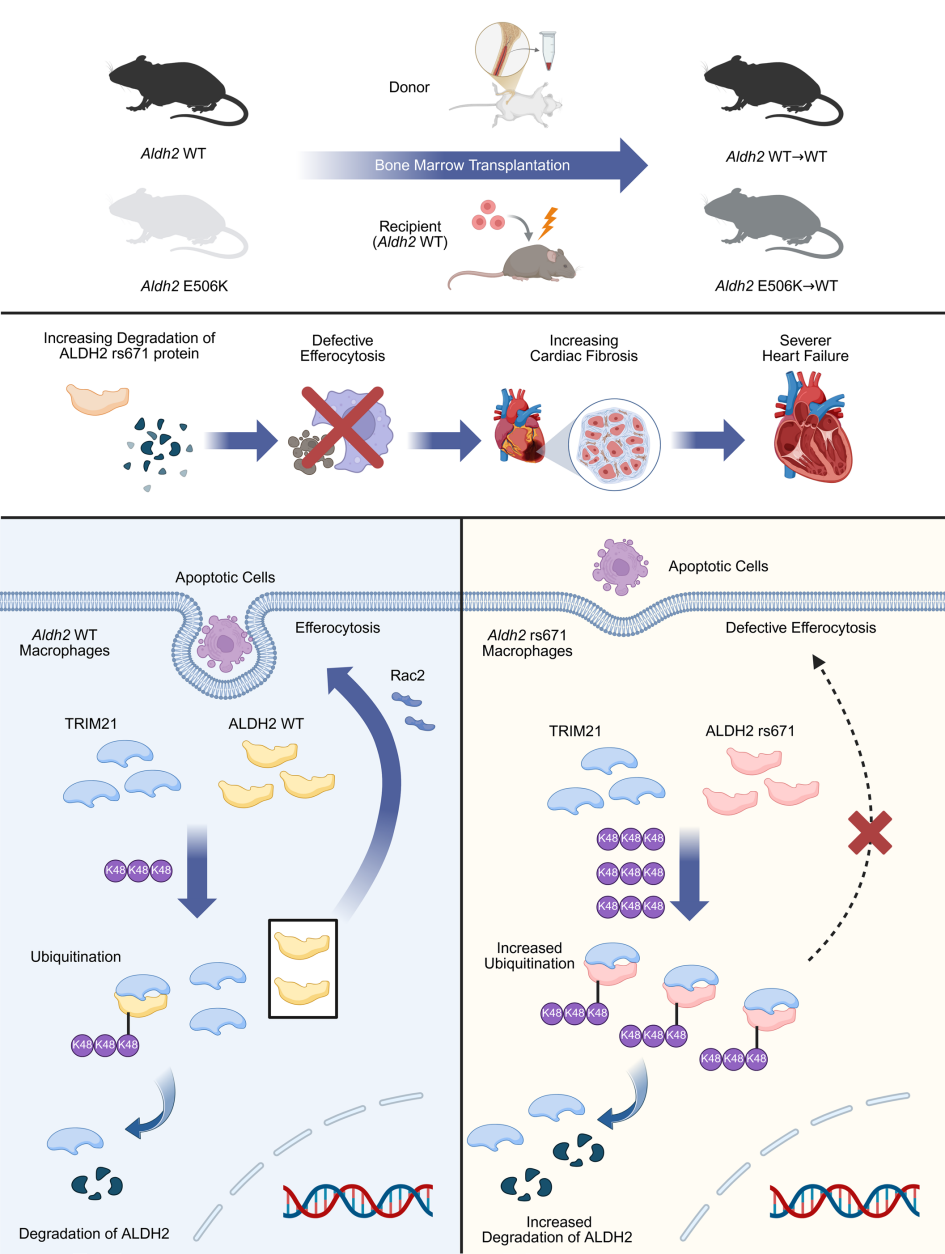
TRIM21 mediated K48-linked ubiquitination of ALDH2 rs671 mutant promotes adverse cardiac remodeling by suppressing efferocytosis. TRIM21 directly interacted with ALDH2 and upregulating ALDH2 rs671 mutant K48-linked ubiquitination. Increasing ALDH2 degradation accelerated Rac2 degradation and resulted in efferocytosis deficiency. Ultimately, *Aldh2* rs671 variant led to adverse cardiac remodeling after MI.

This study demonstrates the ubiquitination regulatory function of the E3 ligase TRIM21 toward ALDH2 (Figure 7). TRIM21 directly binds to and promotes K48-linked ubiquitination of the ALDH2 rs671 mutant. Enhanced binding affinity between TRIM21 and the ALDH2 rs671 protein results in increased ubiquitination and accelerated degradation of ALDH2. Following MI, TRIM21 expression is upregulated, further augmenting its interaction with ALDH2 and promoting ubiquitination of ALDH2. This mechanism may explain why ALDH2 is downregulated after MI. These findings suggest that targeting TRIM21 expression or disrupting its interaction with ALDH2 may offer a therapeutic approach for MI. Collectively, our results provide a convincing explanation for why the ALDH2 rs671 protein shows increased degradation. Additionally, the potential upstream pathway of ALDH2 regulating the ubiquitination of Rac2 has been clarified in this study. Furthermore, the TRIM21-ALDH2-Rac2-efferocytosis axis has been revealed during cardiac fibrosis after MI, which may partially answer previous clinical questions about how the *Aldh2* rs671 variant confers an elevated risk of HF after MI.

Several studies have clarified a potentially novel nonenzymatic function of ALDH2 in cardiovascular diseases. For example, phosphorylated ALDH2 induced by AMPK translocate to the nucleus to regulate the transcription of *Atp6v0e2*, regulating lysosomal function and autophagy and preventing foam cell formation during atherosclerosis (14). Our previous research demonstrated that the mutant ALDH2 rs671 protein showed a reduced interaction with Rac2, resulting in increased ubiquitination of Rac2. Enhanced Rac2 protein degradation subsequently led to impaired efferocytosis in macrophages, thereby accelerating necrotic core formation during atherosclerosis (13). Furthermore, studies focusing on the upstream regulation of ALDH2 are still lacking. We used mass spectrometry–based proteomics and finally identified that E3 ligase TRIM21 interacted with ALDH2.

TRIM21 is a member of the tripartite motif protein (TRIM) family, which consists of more than 80 proteins. The TRIM21 gene, situated on chromosome 11, encodes a ubiquitously expressed protein known as a RING-dependent E3 ligase, which is present in both the cytoplasm and nucleus of cells (15–17). TRIM21 protein is widely recognized for its role in inflammation (18–21), cancer (22–25), and autoimmunity (26–29), including systemic lupus erythematosus (30, 31) and Sjögren’s syndrome (32). Moreover, TRIM21 has been shown to participate in MI. A previous study demonstrated that KO of TRIM21 mitigated MI-induced atrial remodeling by triggering the NF-κB signaling pathway activation, thus promoting Nox2 expression. Upregulation of Nox2 promotes ROS production, oxidative damage, and inflammation, ultimately leading to atrial remodeling (33). In addition, TRIM21 aggravated post-MI cardiac injury by enhancing M1 macrophage polarization (34). More recently, TRIM21 was found to exacerbate hypoxia-induced cardiomyocyte pyroptosis in post-MI HF (35) and to enhance I/R injury by blocking FAK/NF-κB signaling pathway (36). In this study, we revealed an alternative pathway of TRIM21 affecting cardiac fibrosis after MI: the ubiquitination of ALDH2 and defective efferocytosis. Additionally, since our study utilized BM chimeric mice and previous studies have demonstrated that TRIM21 can also regulate other immune cells such as CD4^+^ T cells (37) and CD8^+^ T cells (38), whether TRIM21 affects cardiac remodeling via regulating other immune cells needs further exploration.

The *Aldh2* rs671 variant represents a prevalent genetic variation among the East Asian population (39). Accumulating evidence indicates that ALDH2 confers cardiovascular protection through multiple mechanisms, including ischemic preconditioning (40, 41), autophagy (42), and the renin-angiotensin system (RAS) (43). Our study defines a mechanism by which ALDH2 exerts cardioprotective function by regulating efferocytosis in macrophages. Compared with the rs671 mutant, WT ALDH2 protein is less ubiquitinated by TRIM21, thereby stabilizing Rac2 to ensure continuous efferocytosis. These processes prevent the aggravation of cardiac fibrosis, avoiding enhanced cardiac remodeling.

Macrophages play a pivotal role in mediating post-MI cardiac inflammation. Removal or alteration of these cells during either the proinflammatory or reparative phases of immune response significantly affects the functional recovery of cardiac tissue (44–47). Emerging evidence suggests that the majority of macrophages infiltrating the heart after MI predominantly derive from monocyte precursors originating in hematopoietic tissues (48, 49), while the capacity of resident cardiac macrophages to undergo local proliferation plays a significant role in mediating both adaptive healing and/or maladaptive responses in the injured myocardium (8, 50, 51). Several studies have revealed different functions of macrophages in cardiac remodeling. Deniset et al. found that resident Gata6^+^ macrophages in pericardial fluid played a crucial role in preventing fibrosis of healthy myocardium and improved functional cardiac recovery after ischemic injury (52). Numerous studies have also clarified that shifting the macrophage phenotype from the proinflammatory M1-like macrophage to an antiinflammatory M2-like macrophage protects the heart from adverse remodeling following AMI. In this study, we found that a lack of efferocytosis aggravated cardiac fibrosis and impaired cardiac function, leading to worsening cardiac remodeling. Deficient efferocytosis leads to prolonged inflammation induced by uncleared apoptotic cardiomyocytes and polarization of proinflammatory macrophages, and further it results in excretion of proinflammatory cytokines (such as IL-1β and TNF-α), causing excessive cardiac fibrosis (53, 54).

However, our study has some limitations. We mainly focused on the upstream regulation of ALDH2. The specific downstream mechanisms about how ALDH2 affects cardiac fibrosis through regulating efferocytosis still remain to be investigated. Whether TRIM21 regulating ubiquitination of ALDH2 occurs in other types of cells needs further exploration in the future as well. The certain binding site that TRIM21 interacts on ALDH2 and how the binding site affects the affinity of ubiquitination site on ALDH2 remains to be clarified. Furthermore, since ALDH2 also has its traditional enzymatic functions, we are going to be exploring whether TRIM21-regulating ALDH2 has an influence on post-MI cardiac remodeling through accumulating toxic aldehydes in our following studies.

Additionally, translational medical research targeting TRIM21 and ALDH2 in MI requires further development. Although pharmacological drugs directly targeting ALDH2 are limited, a compound known as fedratinib, has been reported to inhibit TRIM21 expression (55). Our study provides a potential therapeutic role of TRIM21 inhibitors for preventing post-MI HF.

## Methods

### Sex as a biological variable.

Our study examined male and female animals, and similar findings were reported for both sexes. Therefore, sex was not considered as a variable in our study.

### Animals.

We purchased 60 WT mice and 60 *Aldh2* (E506K) mice (4–8 weeks old, initial body weight 20–30 g) from Beijing Viewsolid Biotech Co. Ltd. and housed them in the Laboratory Animal Centre of Qilu Hospital of Shandong University at a density of 5 mice/cage. The environmental conditions were maintained at a temperature of approximately 25°C, a humidity of approximately 50%, a 12-hour day/night cycle, a clean environment, adequate food, and water.

### Patients.

We used the Ficoll density gradient (catalog 17-1440-02, BD Biosciences) to isolate human PBMCs according to the protocols. Then, PBMCs were treated with differentiated medium (RPMI-1640, 10% FBS, 25 ng/mL hM-CSF [human macrophage colony-stimulating factor 1]) for 10 days to differentiate macrophages.

### Induction of MI.

Experimental animals underwent either permanent ligation of the left anterior descending artery (LAD) or sham surgery, following established protocols. In brief, mice were anesthetized via inhalation of 2% isoflurane administered through an isoflurane delivery system. A small 1.2 cm incision was then made on the left chest, and the LAD was permanently ligated using an 8-0 silk suture, placed 2–3 mm below the tip of the left auricle. Animals that did not survive the initial 24-hour postoperative period were excluded from further analysis. Sham-operated mice underwent identical surgical procedures, excluding the LAD ligation. At predetermined time points, mice were euthanized via i.p. injection of 0.1% pentobarbital, followed by tissue collection for subsequent analyses.

### Transthoracic echocardiography.

Cardiac function was evaluated using a Vevo2100 Ultrasound system (VisualSonics) at various postoperative time points. Mice were lightly anesthetized with 0.5% isoflurane and positioned on a temperature-controlled ECG platform. The left ventricle and aortic outflow tract were visualized using 2-dimensional B-Mode imaging, and the sample line was aligned at the maximal cross-sectional area of the left ventricle to guide the acquisition of sequential M-Mode echocardiographic images. Parameters including fractional shortening (FS) and ejection fraction (EF) were quantified from a minimum of 3 separate frames per mouse.

### Immunofluorescence staining and TUNEL assay.

For immunofluorescence analysis, heart tissues were harvested and embedded in optimal cutting temperature (OCT) compound (Sakura). Tissue sections of 7 μm thickness were prepared using a cryostat and subsequently blocked with diluted donkey serum for 1 hour at room temperature (25°C). The sections were then incubated with primary antibodies at 4°C overnight. Signals were visualized with Alexa Fluor–conjugated secondary antibodies (Invitrogen). The primary antibodies used for cell immunofluorescence included: anti-CD68 (ab213363, Abcam, 1:200), anti-Rac2 (60077-1-Ig, ProteinTech, 1:200), anti-TRIM21 (12108-1-AP, ProteinTech, 1:200), and anti-ALDH2 (ab110311, Abcam, 1:200).

### RNA extraction and real-time PCR.

Total RNA was isolated from heart homogenates using TRIzol reagent (Invitrogen) in accordance with the manufacturer’s protocol. Subsequently, 1 μg of RNA from each sample was reverse transcribed into cDNA using a Reverse Transcription Reagent kit (Vazyme). The synthesized cDNA was then amplified through semi-qPCR with SYBR Green Mix (Vazyme).

### Western blotting.

Heart tissues samples and cultured primary macrophages and HEK293Ts (ATCC) were homogenized in lysis buffer containing proteinase and phosphatase inhibitor cocktail. The homogenized samples were run on sodium dodecyl sulfate-polyacrylamide gel electrophoresis (SDS-PAGE) to separate the proteins and transferred to the polyvinylidene difluoride (PVDF) membrane (Merck Millipore). The membranes were blocked at room temperature for 30 minutes with QuickBlock Western Blocking Buffer (Beyotime), after which they were incubated with primary antibodies at 4°C overnight. The membranes were washed with tris-buffered saline-tween-20 (TBST) (Servicebio) 3 times for 5 minutes each and subsequently incubated with secondary antibodies coupled with horseradish peroxidase (HRP) for 90 minutes at room temperature. Following this, the membranes were washed with TBST 3 times for 5 minutes each. Finally, protein blots were detected using an enhanced chemiluminescence (ECL) kit (Merck Millipore) and imaging system (Thermo Fisher Scientific), and the data were analyzed using ImageJ software (NIH). The primary antibodies used for western blotting included: anti-ALDH2 (15310-1-AP, ProteinTech, 1:1,000), anti-TRIM21 (12108-1-AP, ProteinTech, 1:1,000), anti-Ubiquitin (ab134953, abcam, 1:1,000), anti-Flag (14793, 8146, Cell Signaling Technology, 1:1,000), anti-Myc (18583, 2276, Cell Signaling Technology, 1:1,000), anti-HA (3724, 2367, Cell Signaling Technology, 1:1,000), anti-V5 (14440-1-AP, ProteinTech, 1:1,000), and anti-β-actin (66009-1-Ig, ProteinTech, 1:1,000).

### Co-IP.

HEK-293T cells were collected after culturing in T25 culture flasks and were then fully lysed by incubation with NP-40 lysis solution (Beyotime) for 1 hour at 4°C on the rotating mixer. The supernatant was separated by centrifugation at 14,000*g* for 15 minutes at 4°C, and the appropriate amount of primary antibody was added and incubated overnight at 4°C. Subsequently, PureProteome Protein A/G Mix Magnetic Beads (Merck Millipore) were added to the supernatant and incubated at 4°C for 3 hours to make the proteins bind to the magnetic beads. Following this, the magnetic beads were separated by the magnetic rack, and then the samples were separated from the magnetic beads by adding PBS-diluted SDS-PAGE loading buffer (4×) (Proteintech) to the beads and incubating at 100°C for 10 minutes. Ultimately, the protein samples were subjected to SDS-PAGE, and the subsequent steps were identical to those employed in Western blotting. The data were analyzed using ImageJ software. The primary antibodies utilized for Co-IP are: anti-Flag (14793, 8146, Cell Signaling Technology, 1:100), anti-Myc (18583, 2276, Cell Signaling Technology, 1:100), and anti-ALDH2 (15310-1-AP, ProteinTech, 1:100).

### Isolation and culture of adult mouse primary macrophages.

Mice were anesthetized using isoflurane (4% isoflurane with 0.2 L O_2_/min) and subsequently euthanized via cervical dislocation. BMDMs were generated as previously described (56). In brief, BM cells were extracted from the tibia and femurs of 6- to 8-week-old WT or *Aldh2* rs671 mice. These cells were cultured in DMEM supplemented with 10% FBS (Gibco) and 1% penicillin-streptomycin (Gibco), and stimulated with 25 ng/mL mouse M-CSF (macrophage colony-stimulating factor 1; BioLegend) for 6 days. Peritoneal macrophages were isolated from ALDH2^–/–^ and C57BL/6J mice 3 days after i.p. injection of a 4% thioglycolate solution (Sigma-Aldrich), following established protocols (57).

### BM transplantation.

The mice were lethally irradiated with an 8.5 Gy X-ray dose, followed by i.v. injection (via the tail vein) of 1 × 10^7^ BM cells from donor mice for BM transplantation, as previously described (56). Briefly, mice were fasted and administered DMEM culture medium intragastrically 1 day before and 2 days after transplantation. During the 4-week recovery phase, mice were provided with water containing sulfamethoxazole (200 mg/mL) and trimethoprim (40 mg/mL). After recovery, the mice underwent LAD ligation. BM donors included WT mice and *Aldh2* rs671 mice, which were used to reconstitute the lethally irradiated WT mice.

### Efferocytosis assay.

For the in vitro efferocytosis assay, mouse cardiac myocytes HL-1 (ScienCell), primary peritoneal macrophages, and BMDMs were isolated and cultured. Cells were grown to approximately 80% confluence before use in experiments. HL-1 cells were stained with CellTracker Deep Red dye (Invitrogen, C34565) at a 1:1,000 dilution and then treated with 1 μmol/L staurosporine for 6 hours to induce apoptosis. The apoptotic cardiac myocytes were subsequently scraped and counted. Following cell counting, the apoptotic cells were cocultured with peritoneal macrophages or BMDMs at a 5:1 ratio for 90 minutes. After removing unbound apoptotic cells by washing, the macrophages were collected and subjected to immunofluorescence staining.

### Plasmids and AAV construction.

The full-length cDNAs of ALDH2 and its mutants (rs671, truncation, and single lysine site), TRIM21, TRIM28, NEDD4, and ubiquitin and its K48/K63 mutants were amplified by standard PCR. ALDH2 and its mutants were tagged with Flag, TRIM21 and TRIM28 were tagged with Myc, NEDD4 was tagged with V5, and ubiquitin and its K48/K63 mutants were tagged with HA and subcloned into pcDNA 3.1 vector, while pcDNA 3.1 plasmid was used as controls. The experimental subgroups were designed in order: the Flag-ALDH2 group, the Myc-TRIM21 group, the Myc-TRIM28 group, the V5-NEDD4 group, and the HA-Ubiquitin group. All constructed plasmids were transfected into HEK-293T cells using Lipofectamine 2000 (Invitrogen) according to the manufacturer’s instructions. The AAV9 vectors targeting the mouse ALDH2 and TRIM21 promoter were all synthesized by Shandong WZ Bioscience Co. Ltd. The interfering sequence designed for TRIM21 gene was tandemly inserted into the multiple cloning site (MCS) of the vector in the form of miR30 structure with CD68-specific promoter. The vector elements were as follows: pUC-CD68-RFP-miR30(MCS)-SV40 PolyA. *Trim21* shRNA sequence is as follows: 5′-GAGCCTATGAGTATCGAAT-3′. The titration of AAV-*Aldh2* is 1 × 10^13^ vg/mL, and the titration of AAV-sh*Trim21* is 6.75 × 10^13^ vg/mL. Each 8-week-old mice were injected 5 × 10^11^ vg AAV via the tail vein.

### Statistics.

All data were assessed for normality and homogeneity of variance. Data that met the normality assumption were expressed as mean ± SEM and analyzed using either the 2-tailed Student’s *t* test or 1-way ANOVA with Tukey’s post hoc test. For data that did not satisfy the normality assumption, results were presented as median (interquartile range) and analyzed using either the Mann-Whitney *U* test or Kruskal-Wallis test, followed by Dunn’s post hoc test. All statistical analyses were performed using GraphPad Prism 9 software (GraphPad). No formal power analysis was conducted to determine sample size, and no data points were excluded from the analysis. A *P* value of less than 0.05 was considered statistically significant.

### Study approval.

All animal procedures performed conform to guidelines from Animal Research: Reporting of In Vivo Experiments. All animal and human studies was approved by the Medical Institutional Ethics Committee of Qilu Hospital, Shandong University, China. For human studies, written informed consent was received prior to participation.

### Data availability.

All data related to this study’s findings are included in this article and its Supporting Data Values file. The supplemental data of mass spectrum of Figure 3A are included in Supplemental Data.

## Author contributions

All authors listed contributed to this article. TH performed most of the experiments and wrote manuscript. XW performed part of molecular biology experiments and animal experiments. XZ provided technical support for molecular experiments. JZ and YG performed the study design and reviewed the manuscript. FX and YC provided resources, critical suggestions, and supervision and critically reviewed the manuscript. All authors read and approved the final manuscript.

## Funding support

- State Key Program of the National Natural Science Foundation of China (82302466, 82325031, 82030059).
- National Natural Science Regional Innovation Fund joint fund key support projects (U23A20485).
- Key R&D Program of Shandong Province (2022ZLGX03).
- National Distinguished Physician Program, Taishan Pandeng Scholar Program of Shandong Province (tspd20240819).
- Youth Program of Natural Science Foundation of Shandong Province (ZR2023QH220).
- Taishan Scholar Young Expert Foundation (tsqn202312329).

## Supplementary Material

Supplemental data

Supplemental Data of Mass Spectrum Results of ALDH2

Unedited blot and gel images

Supporting data values

## Version 1 02/24/2026

In-Press Preview

## Version 2 04/08/2026

Electronic publication
