# Supplementary material for: E3 ubiquitin ligase TRIM21-mediated K48-linked ubiquitination of ALDH2 rs671 mutant promotes adverse cardiac remodeling
Source: JCI Insight. 2026 Feb 24;11(7):e197555. doi: 10.1172/jci.insight.197555 (PMC13134731; doi:10.1172/jci.insight.197555)
Supplement: Supplemental data [file jciinsight-11-197555-s149.pdf]

Supplementary Materials

E3 ubiquitin ligase TRIM21-mediated K48-linked ubiquitination of ALDH2 rs671 mutant promotes adverse cardiac remodeling

Tianrui Han[1,2,3,4,5], Xin Wen[1,2,3,4,5], Yunyun Guo[1,2,3,4,5], Xiangkai Zhao[1,2,3,4,5], Jian Zhang[1,2,3,4,5], Yuguo Chen[1,2,3,4,5], Feng Xu[1,2,3,4,5]

1. Department of Emergency Medicine, Qilu Hospital of Shandong University, Jinan, China

2. Shandong Provincial Clinical Research Center for Emergency and Critical Care Medicine, Institute of Emergency and Critical Care Medicine of Shandong University, Chest Pain Center, Qilu Hospital of Shandong University, Jinan, China

3. Medical and Pharmaceutical Basic Research Innovation Center of Emergency and Critical Care Medicine, China's Ministry of Education, Shandong Provincial Engineering Laboratory for Emergency and Critical Care Medicine, Key Laboratory of Emergency and Critical Care Medicine of Shandong Province, Key Laboratory of Cardiopulmonary-Cerebral Resuscitation Research of Shandong Province, Qilu Hospital of Shandong University, Jinan, China

4. NMPA Key Laboratory for Clinical Research and Evaluation of Innovative Drug, Qilu Hospital of Shandong University, Jinan, China

5. State Key Laboratory for Innovation and Transformation of Luobing Theory; Key Laboratory of Cardiovascular Remodeling and Function Research, Chinese Ministry of Education, Chinese National Health Commission and Chinese Academy of Medical Sciences, Qilu Hospital of Shandong University, Jinan, China

Address correspondence to: Feng Xu or Yuguo Chen or Jian Zhang, No. 107, Wen Hua Xi Road, Qilu Hospital of Shan-dong University, Jinan, Shandong 250012, China. Phone: (+86) 0531-82166844; Email: xufengsdu@126.com (FX); chen919085@sdu.edu.cn (YC); 18366119306@163.com (JZ).

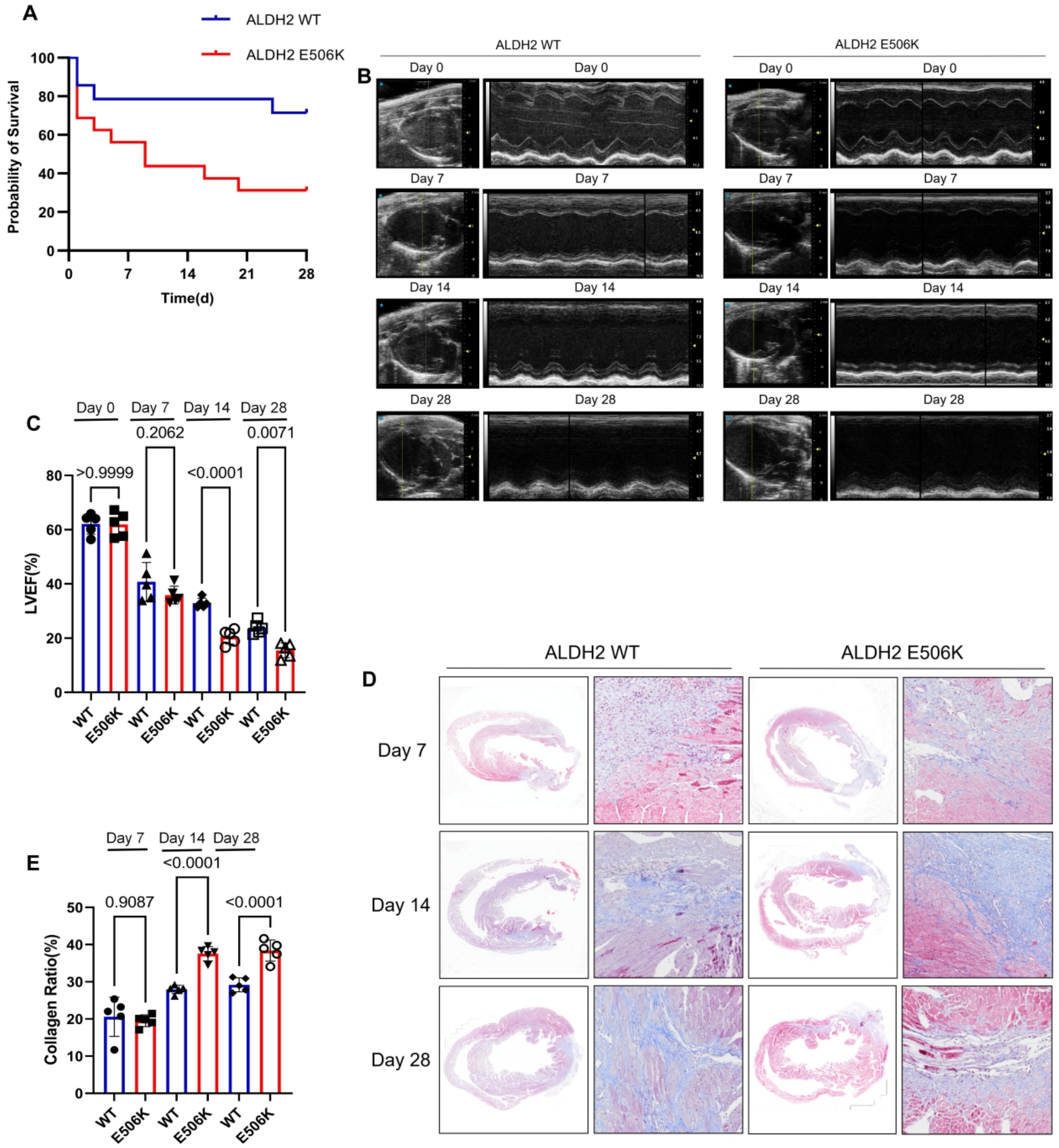

Figure S1. ***Aldh2*** **rs671 mutation leads to aggravating cardiac fibrosis.** **A** Probability of survival curve of wild-type (WT) mice and rs671 mice after MI operation. **B and C** Representative parasternal long-axis views and M-mode images. Echocardiographic analysis of ejection fraction (EF) on days 0, 7, 14, 28 after MI in WT and rs671 mice.(n=5) **D and E** Representative Masson trichrome staining of cardiac tissue obtained from WT and rs671 mice on days 7, 14, 28 after MI. Quantitative analysis of

collagen ratio on day 7, 14, 28 after MI in WT and rs671 mice.(n=5) Data are expressed as mean ± SEM. One-way ANOVA and Tukey post hoc test were used for analysis.

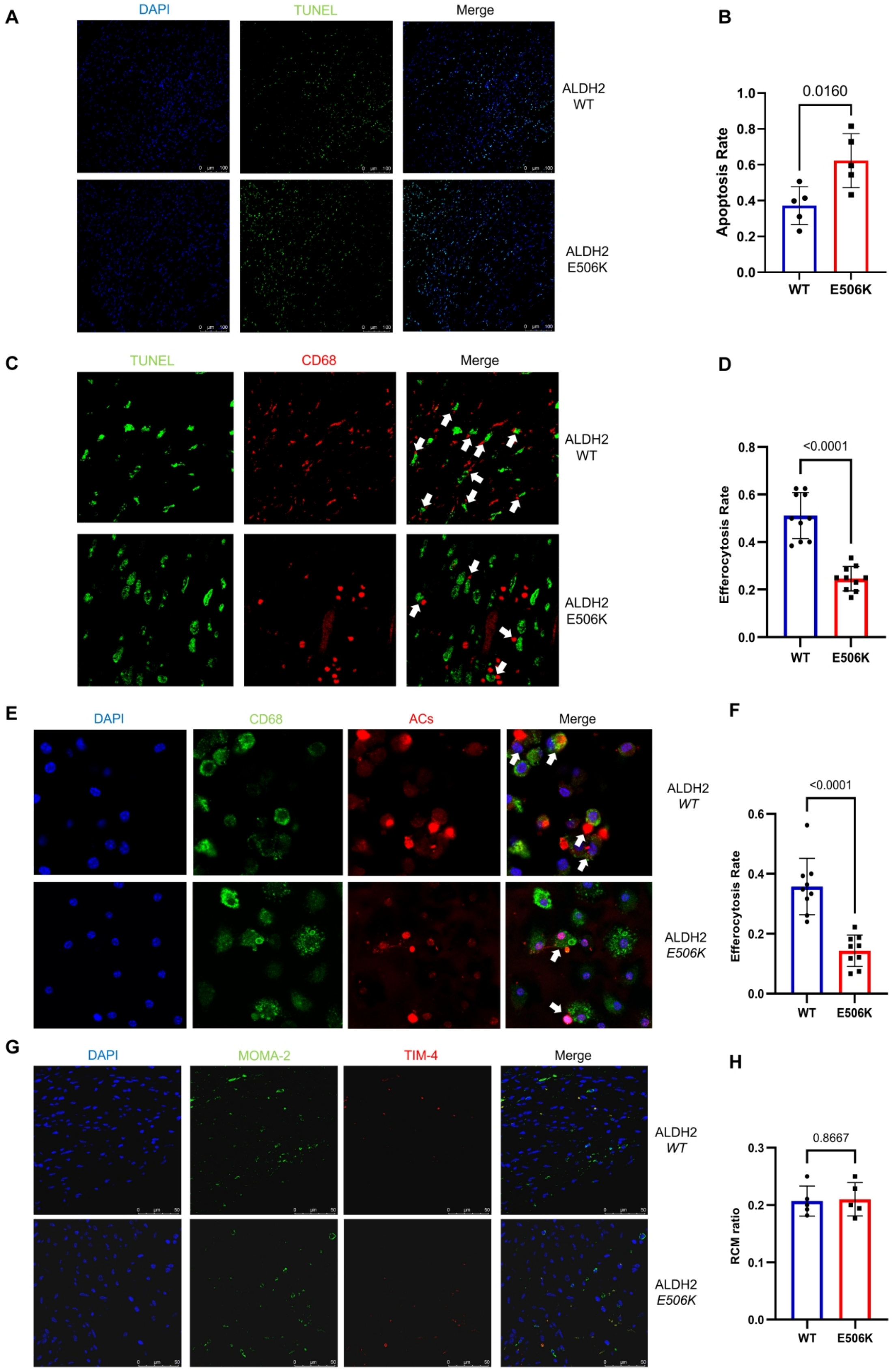

Figure S2. *Aldh2* rs671 macrophages have a defect in efferocytosis of

apoptotic cardiomyocytes. A and B Representative photomicrographs of terminal deoxynucleotidyl transferase dUTP nick-end labeling (TUNEL, green) staining with nuclear DAPI (blue) in cardiac tissue obtained from WT and rs671 mice on day 14 after MI operation. Analysis of apoptosis rate in heart tissue after MI.(n=5) **C and D** Representative photomicrographs of terminal deoxynucleotidyl transferase dUTP nick-end labeling (TUNEL, green) staining with macrophages marker CD68 (red) in cardiac tissue obtained from WT and rs671 mice on day 14 after MI operation. Analysis of internalization of apoptotic cardiomyocytes in macrophages. Macrophages that locate close to apoptotic cells are scored as having internalized apoptotic cardiomyocytes.(n=10) **E and F** Immunohistochemistry shows colocalization of CD68+ BMDMs (green) with apoptotic cardiomyocytes (red). Percentage of apoptotic cells-associated macrophages, compared between WT and rs671 mice BMDMs.(n=9) **G and H** Representative photomicrographs of TIM-4 (red) and MOMA-2 (green) staining with nuclear DAPI (blue) in cardiac tissue obtained from WT and rs671 mice on day 14 after MI operation. Analysis of relative TIM-4 expression in macrophages after MI.(n=5) Data are expressed as mean ± SEM. Unpaired two-tailed Student's t-test were used for analysis.

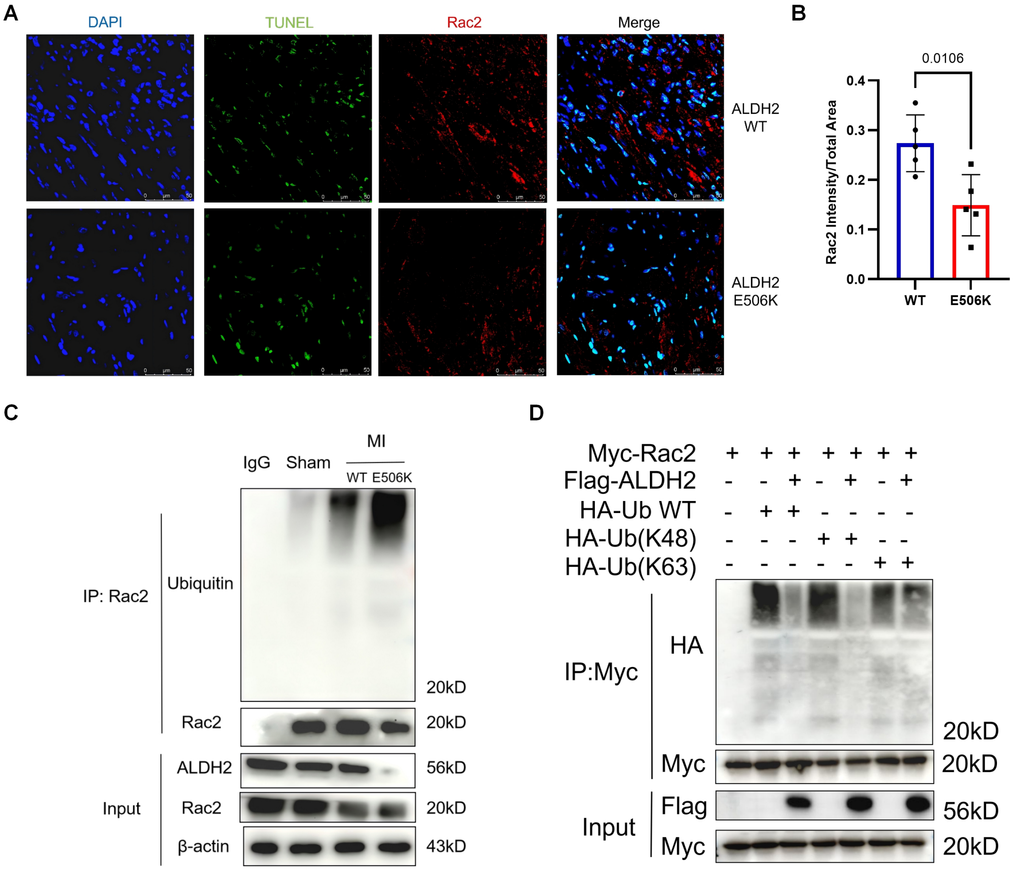

Figure S3. *Aldh2* rs671 variant downregulates Rac2 expression by increasing Rac2 protein ubiquitination level. A and B Representative photomicrographs of Rac2 (red) and TUNEL (green) staining with nuclear DAPI (blue) in cardiac tissue obtained from WT and rs671 mice on day 14 after MI operation. Analysis of Rac2 expression in macrophages after MI.(n=5) **C** Co-IP using anti-Rac2 antibody was performed with lysates from *Aldh2* rs671 or wild-type mice BMDMs treated with or without MI surgery followed by Western blotting. β-actin were used as loading controls. **D** HA-ubiquitin, HA-ubiquitin(K48) and HA-ubiquitin(K63) were co-transfected into HEK293T cells with

Flag-ALDH2 and Myc-Rac2. Cell lysates were subjected to Co-IP with anti-Myc antibody and followed by Western blotting.

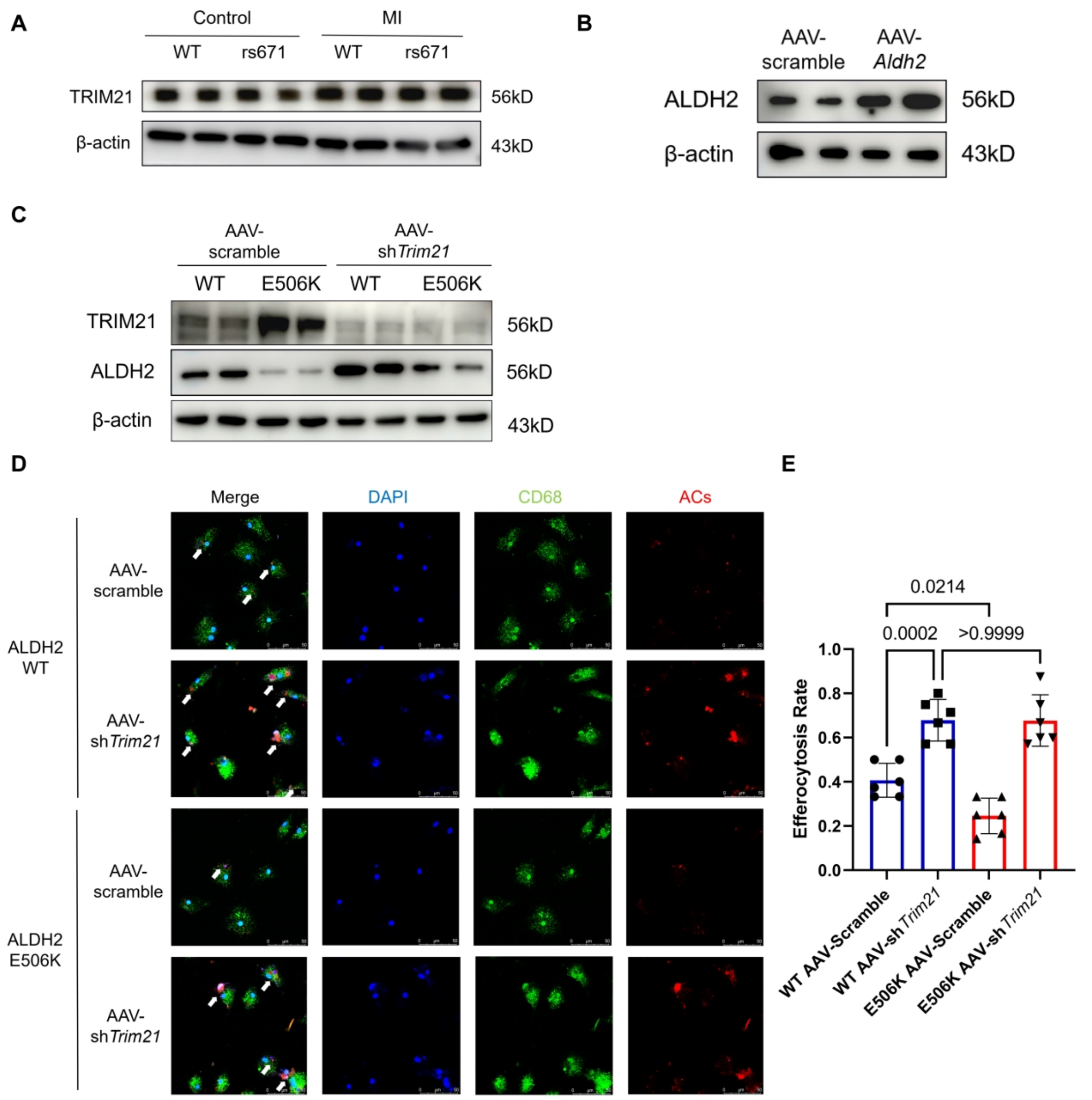

Figure S4. Overexpressing *Aldh2* and downregulating *Trim21* enhances efferocytosis impaired by *Aldh2* rs671 mutation. A Representative bands of TRIM21 protein level was obtained from PBMCs of *Aldh2* WT and rs671 individuals with or without MI. **B** Representative bands of ALDH2 protein level was obtained from peritoneal macrophages of ALDH2 WT and rs671 mice injected with AAV-Scramble or AAV-*Aldh2*. **C** Representative bands of TRIM21 protein level was obtained from peritoneal macrophages of ALDH2 WT

and rs671 mice injected with CD68-promoter AAV-Scramble or AAV-sh*Trim21*. **D and E** Immunohistochemistry shows colocalization of CD68+ macrophages (green) with TUNEL+ cardiomyocytes (red). Percentage of TUNEL-positive macrophages, compared between WT and rs671 mice.(n=6) β-actin were used as loading controls. Data are expressed as mean ± SEM. One-way ANOVA and Tukey post hoc test were used for analysis.

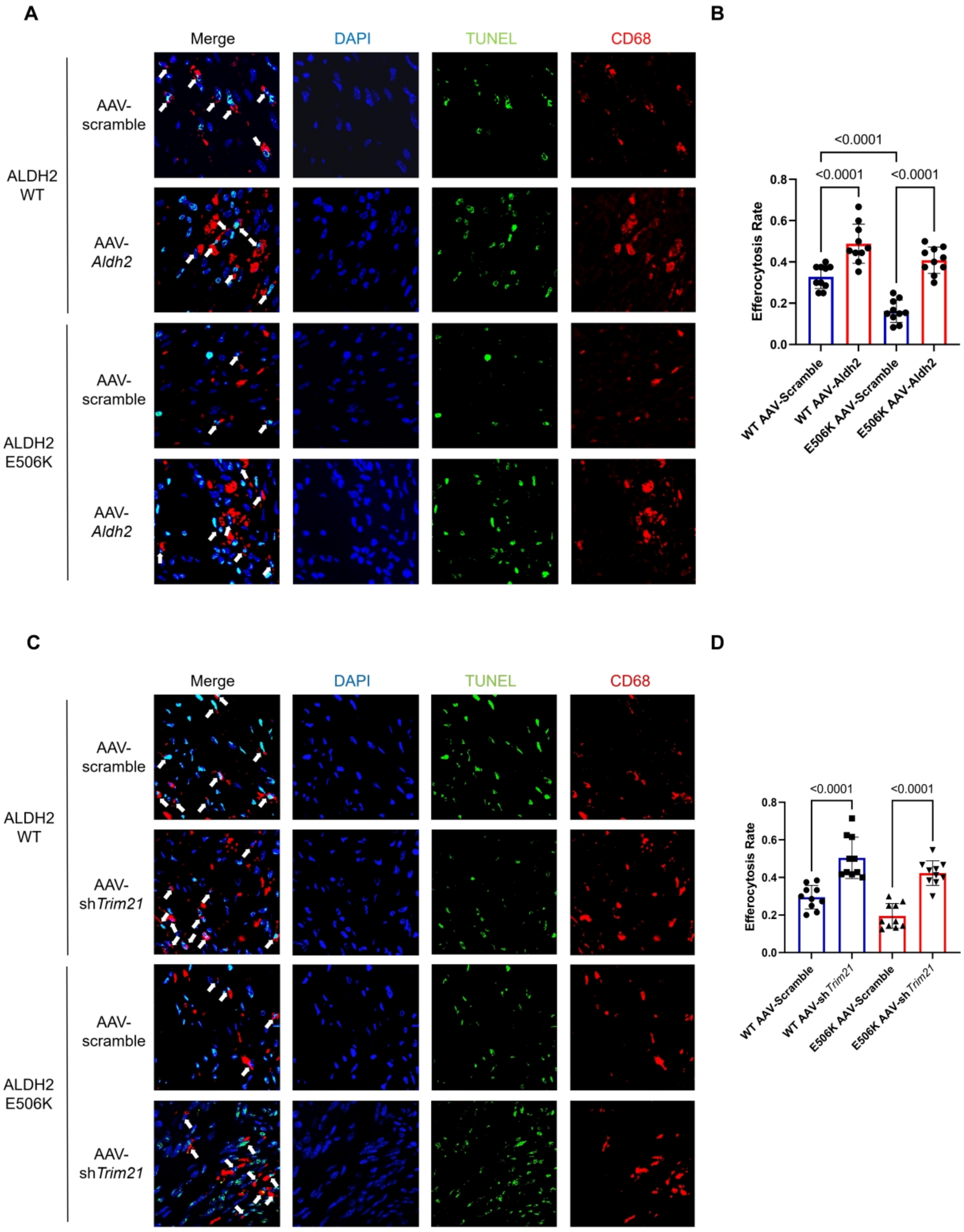

Figure S5. Overexpressing *Aldh2* and downregulating *Trim21* rescues deficient efferocytosis in cardiac tissue after MI caused by *Aldh2* rs671 variant. A and B Representative photomicrographs of TUNEL (green) and nuclear DAPI (blue) staining with macrophages marker

CD68 (red) in cardiac tissue obtained from WT and rs671 mice injected with AAV-scramble or AAV-*Aldh2* on day 14 after MI operation. Analysis of internalization of apoptotic cardiomyocytes in macrophages. Macrophages that locate close to apoptotic cells are scored as having internalized apoptotic cardiomyocytes.(n=10) **C and D** Representative photomicrographs of TUNEL (green) and nuclear DAPI (blue) staining with macrophages marker CD68 (red) in cardiac tissue obtained from WT and rs671 mice injected with CD68-promoter AAV-scramble or AAV-sh*Trim21* on day 14 after MI operation. Analysis of internalization of apoptotic cardiomyocytes in macrophages. Macrophages that locate close to apoptotic cells are scored as having internalized apoptotic cardiomyocytes.(n=10) Data are expressed as mean ± SEM. One-way ANOVA and Tukey post hoc test were used for analysis.
