# Supplementary material for: E3 ubiquitin ligase TRIM21-mediated K48-linked ubiquitination of ALDH2 rs671 mutant promotes adverse cardiac remodeling
Source: JCI Insight. 2026 Feb 24;11(7):e197555. doi: 10.1172/jci.insight.197555 (PMC13134731; doi:10.1172/jci.insight.197555)
Supplement: Supplemental Data of Mass Spectrum Results of ALDH2 [file jciinsight-11-197555-s150.zip › Q62191-GSELELLQEVR-PD.by.data.pdf]

label	mz	intensity
b2(1+)−H2O	127.05	24697.2
y2(2+)−NH3	129.10	18518.8
b2(1+)	145.06	12949.8
y1(1+)−NH3	158.09	3515.7
y1(1+)	175.12	33274.7
b4(2+)−H2O	185.09	3909.4
b3(1+)−H2O	256.09	33848.8
y2(1+)−NH3, y (4) (2+)−H2O	257.16	7243.6
b3(1+)	274.10	17625.4
y2(1+)	274.19	29750.2
b4(1+)−H2O	369.18	5146.0
b4(1+)	387.19	5873.4
y3(1+)	403.23	7999.1
b5(1+)−H2O	498.23	4184.3
y4(1+)	531.29	22328.0
y5(1+)	644.37	34285.7
y6(1+)	757.46	34450.1
y7(1+)−H2O	868.49	3660.6
y7(1+)	886.50	30621.7
y8(1+)	999.58	9632.9
