# Supplementary figures and images for: E3 ubiquitin ligase TRIM21-mediated K48-linked ubiquitination of ALDH2 rs671 mutant promotes adverse cardiac remodeling
Source: JCI Insight. 2026 Feb 24;11(7):e197555. doi: 10.1172/jci.insight.197555 (PMC13134731; doi:10.1172/jci.insight.197555)

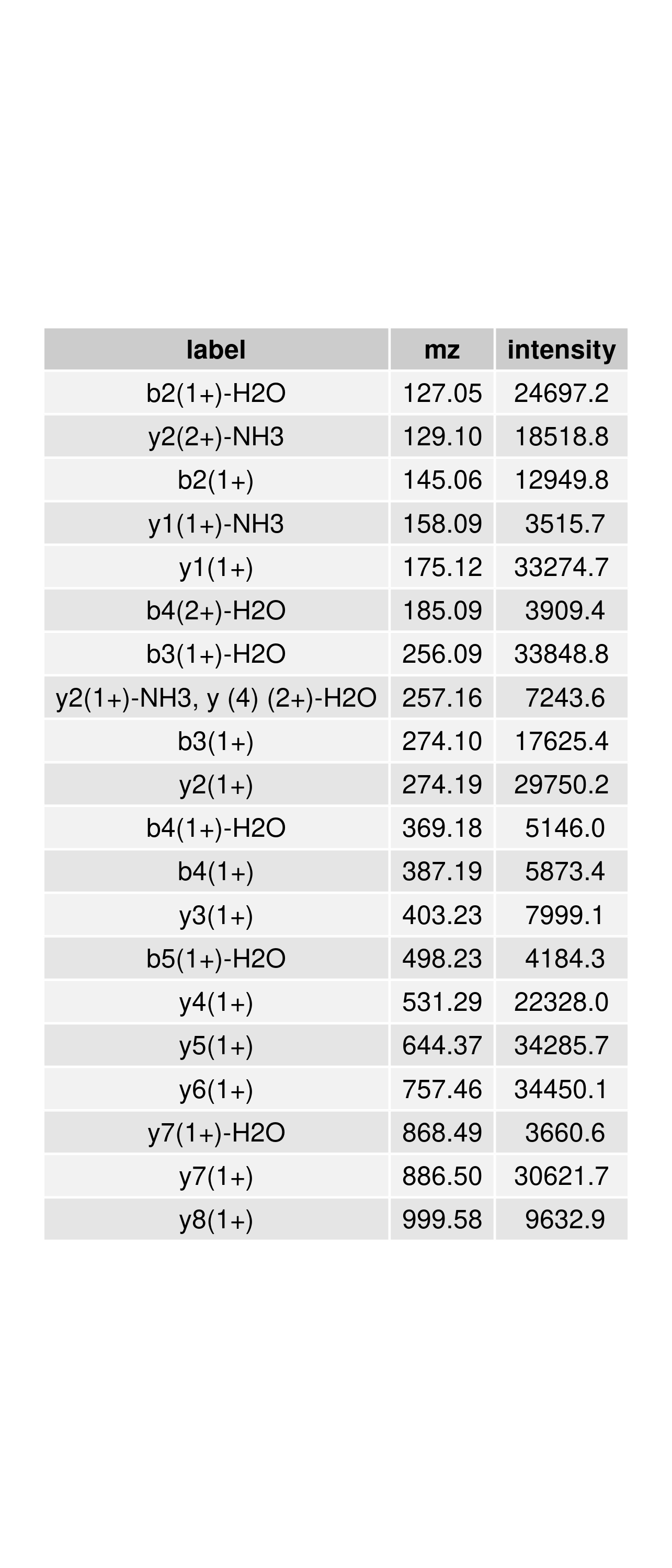

Supplement: Supplemental Data of Mass Spectrum Results of ALDH2 [file jciinsight-11-197555-s150.zip › Q62191-GSELELLQEVR-PD.by.data.png]

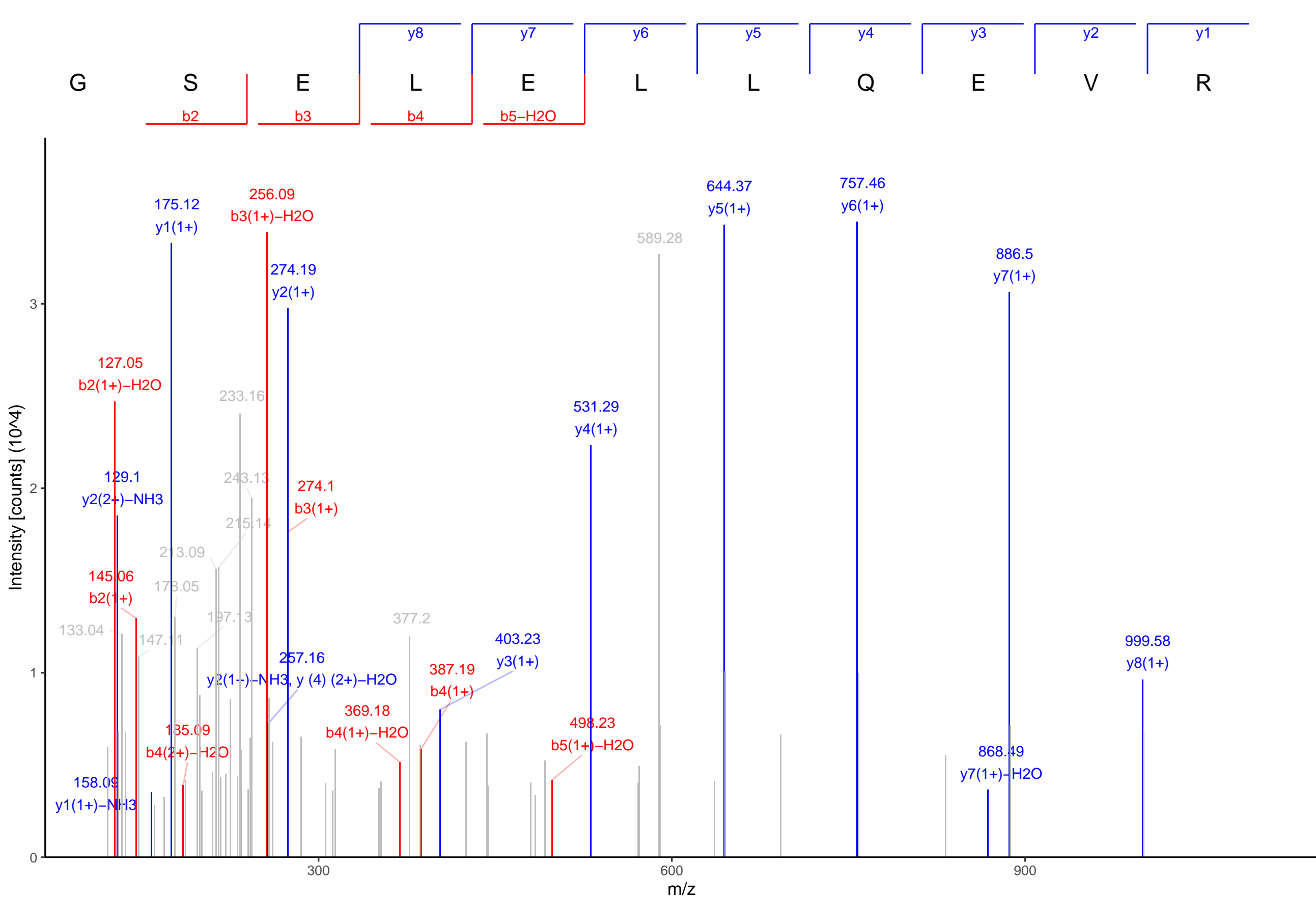

Supplement: Supplemental Data of Mass Spectrum Results of ALDH2 [file jciinsight-11-197555-s150.zip › Q62191-GSELELLQEVR-PD.spectrum.pdf]

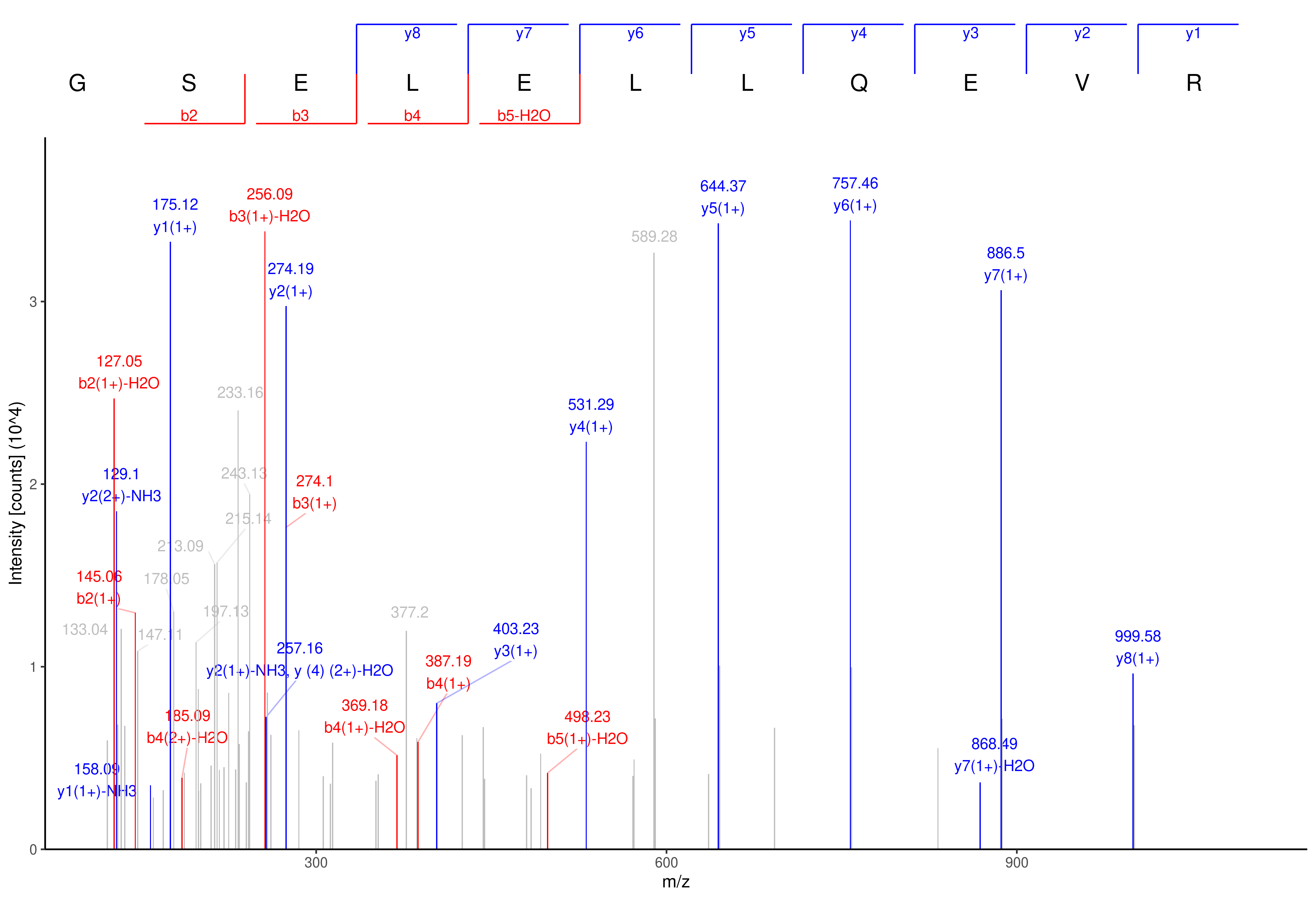

Supplement: Supplemental Data of Mass Spectrum Results of ALDH2 [file jciinsight-11-197555-s150.zip › Q62191-GSELELLQEVR-PD.spectrum.png]
