# Supplementary material for: E3 ubiquitin ligase TRIM21-mediated K48-linked ubiquitination of ALDH2 rs671 mutant promotes adverse cardiac remodeling
Source: JCI Insight. 2026 Feb 24;11(7):e197555. doi: 10.1172/jci.insight.197555 (PMC13134731; doi:10.1172/jci.insight.197555)

Figure 2

A

ALDH2

βactin

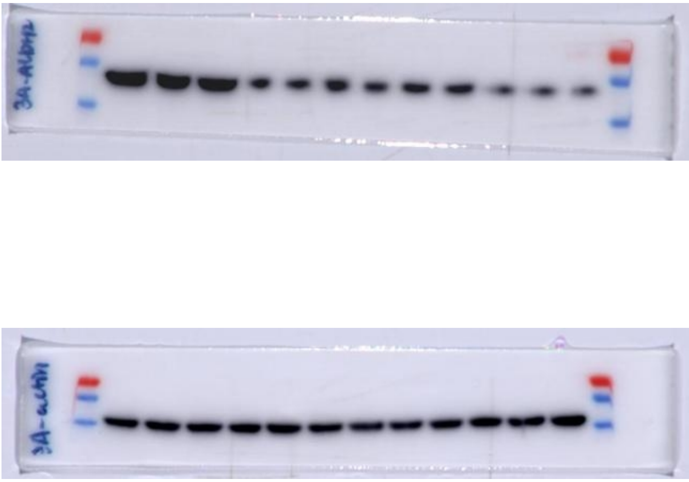

D

ALDH2

βactin

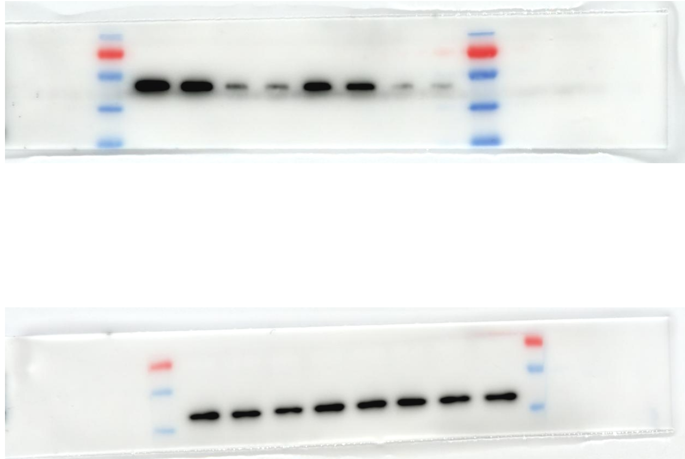

Figure 2

G

ALDH2

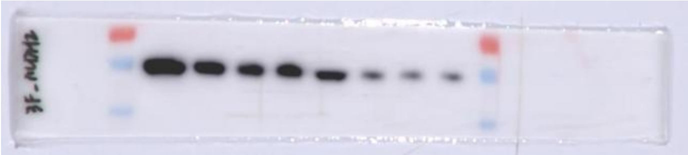

βactin

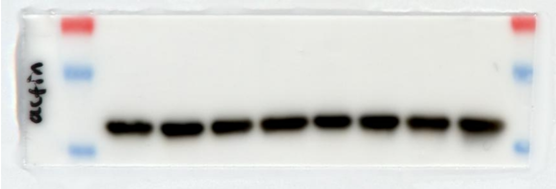

I

ALDH2

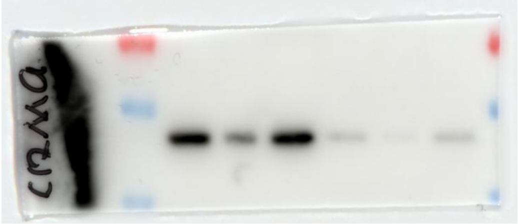

βactin

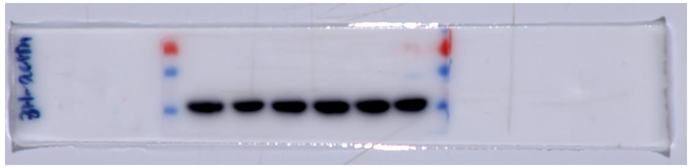

Figure 3

B

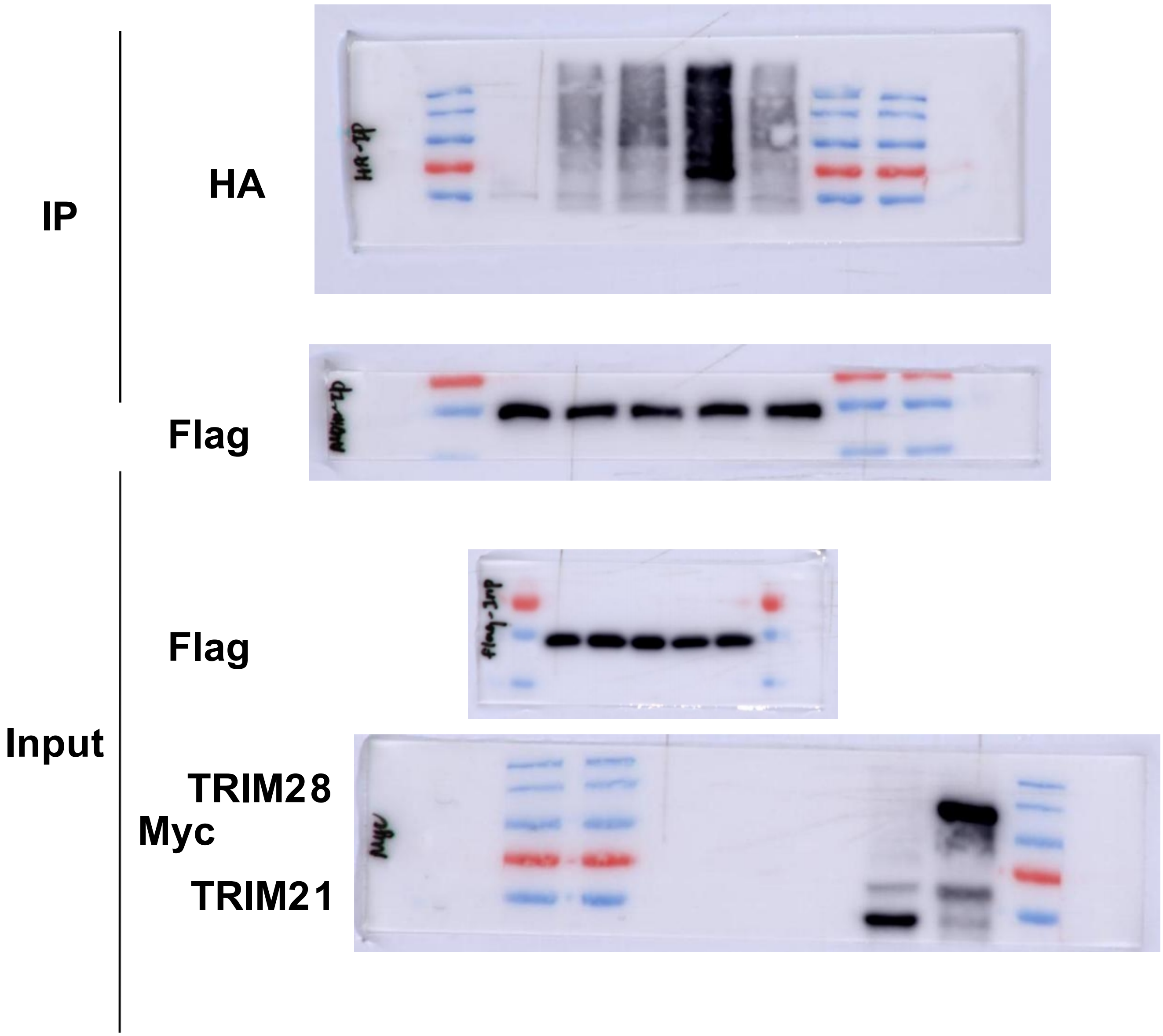

Figure 3

C

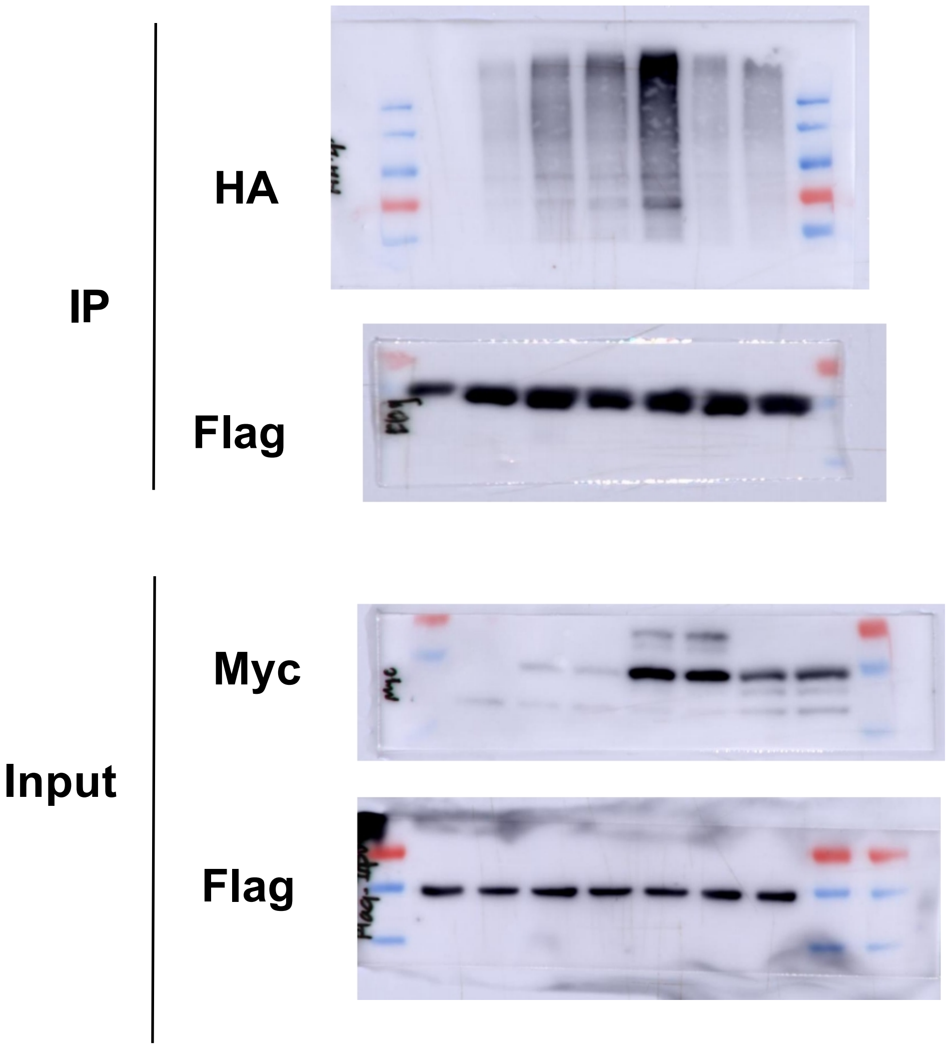

Figure 3

D

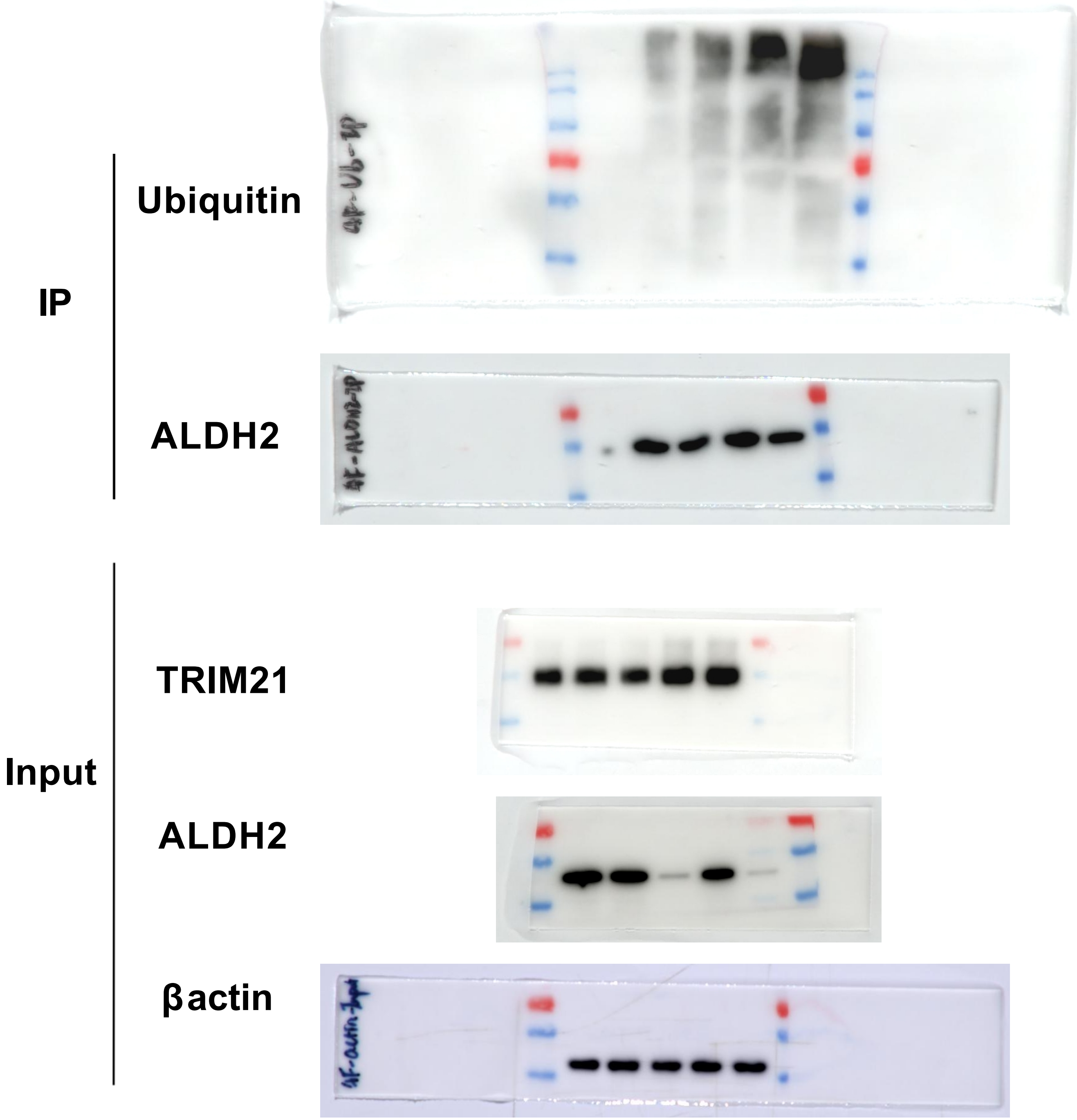

Figure 3

E

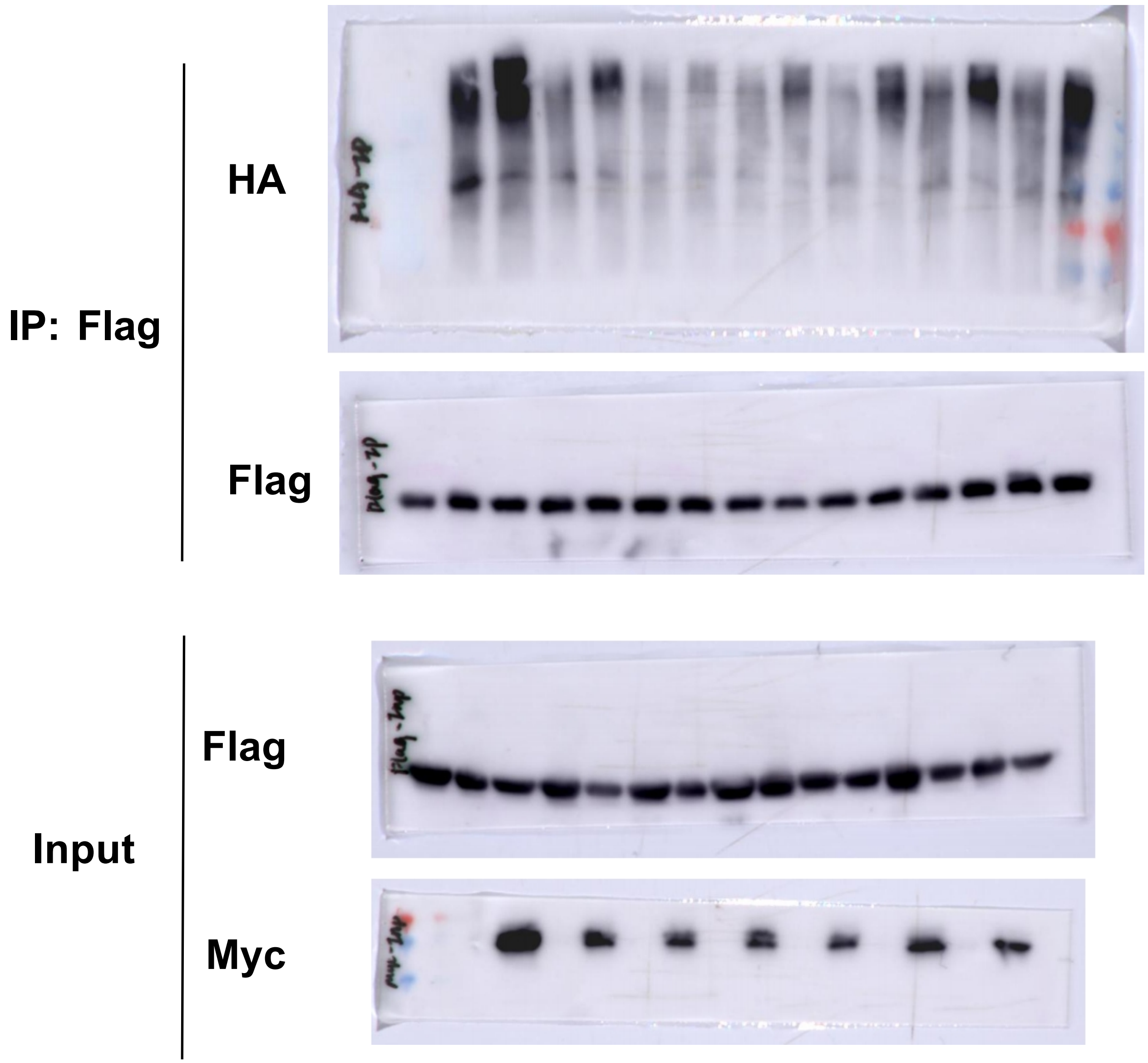

Figure 3

F

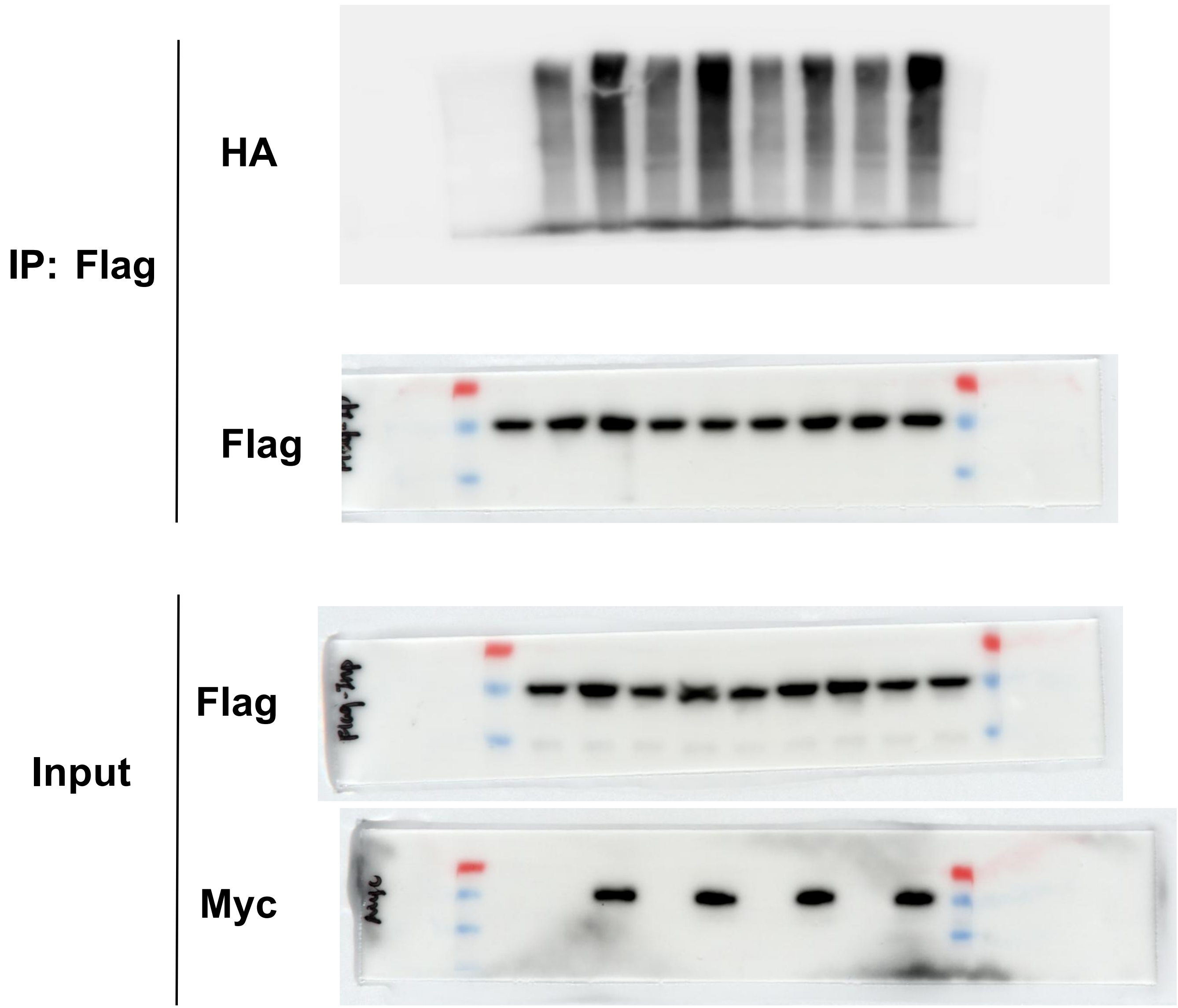

Figure 3

G

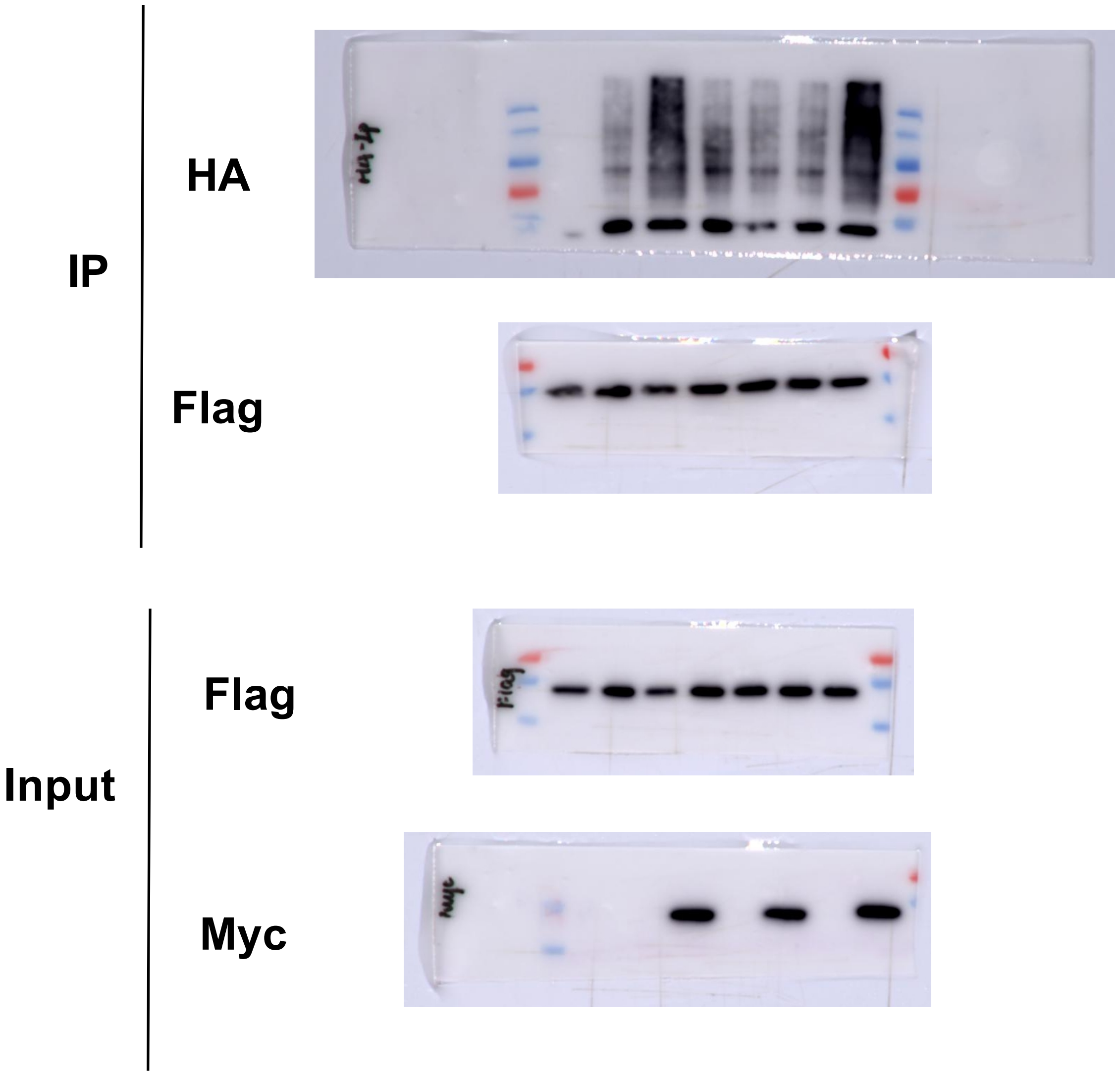

Figure 3

H

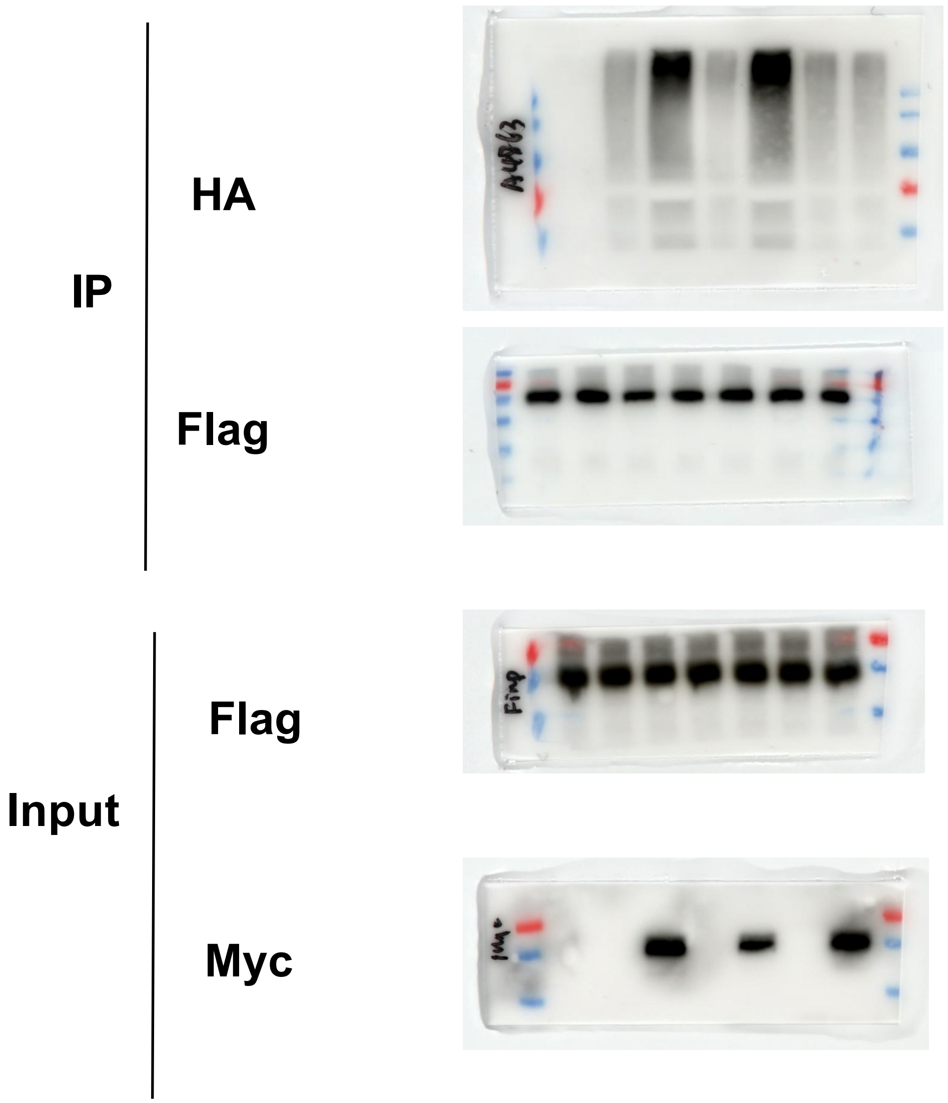

Figure S3

C

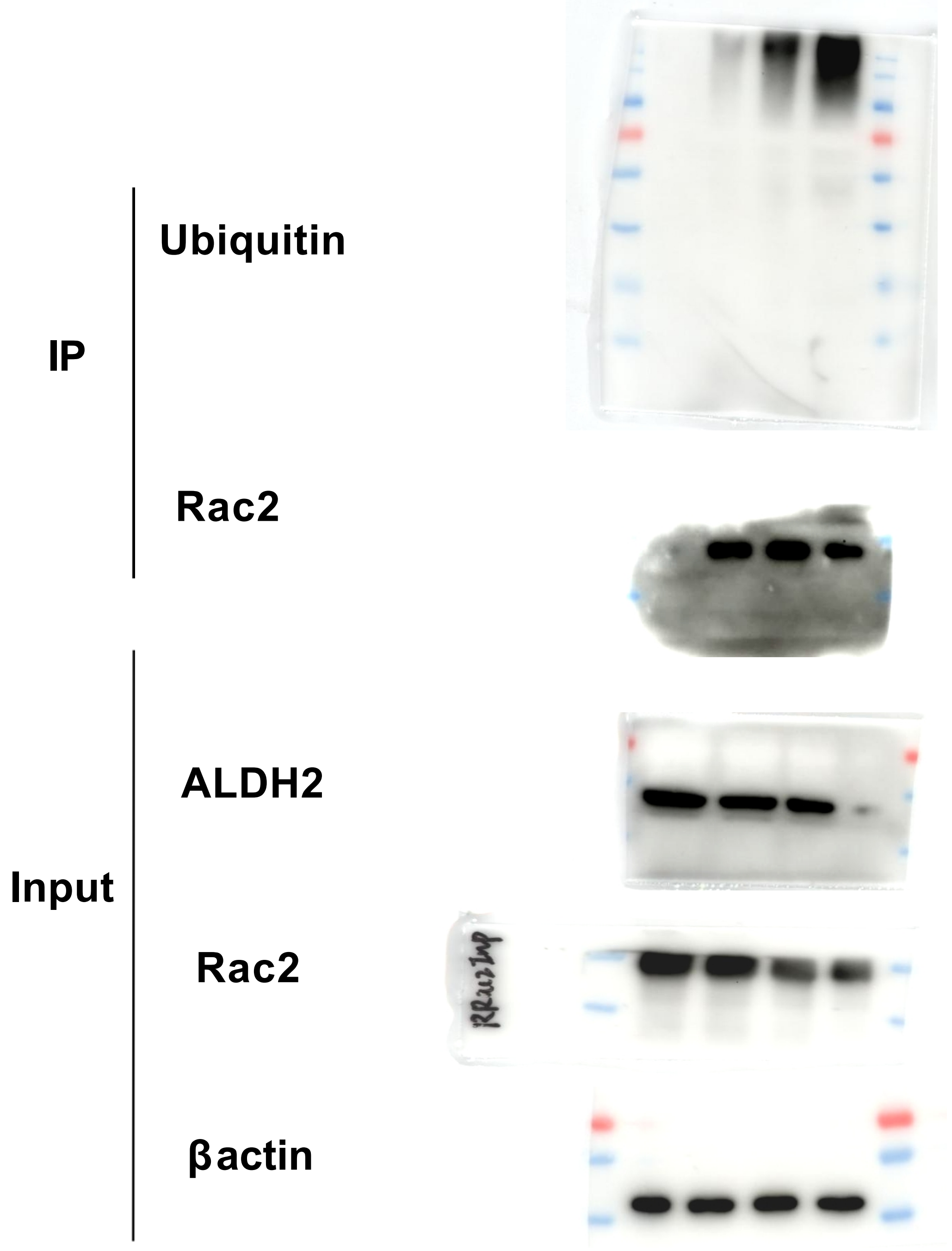

Figure S3

D

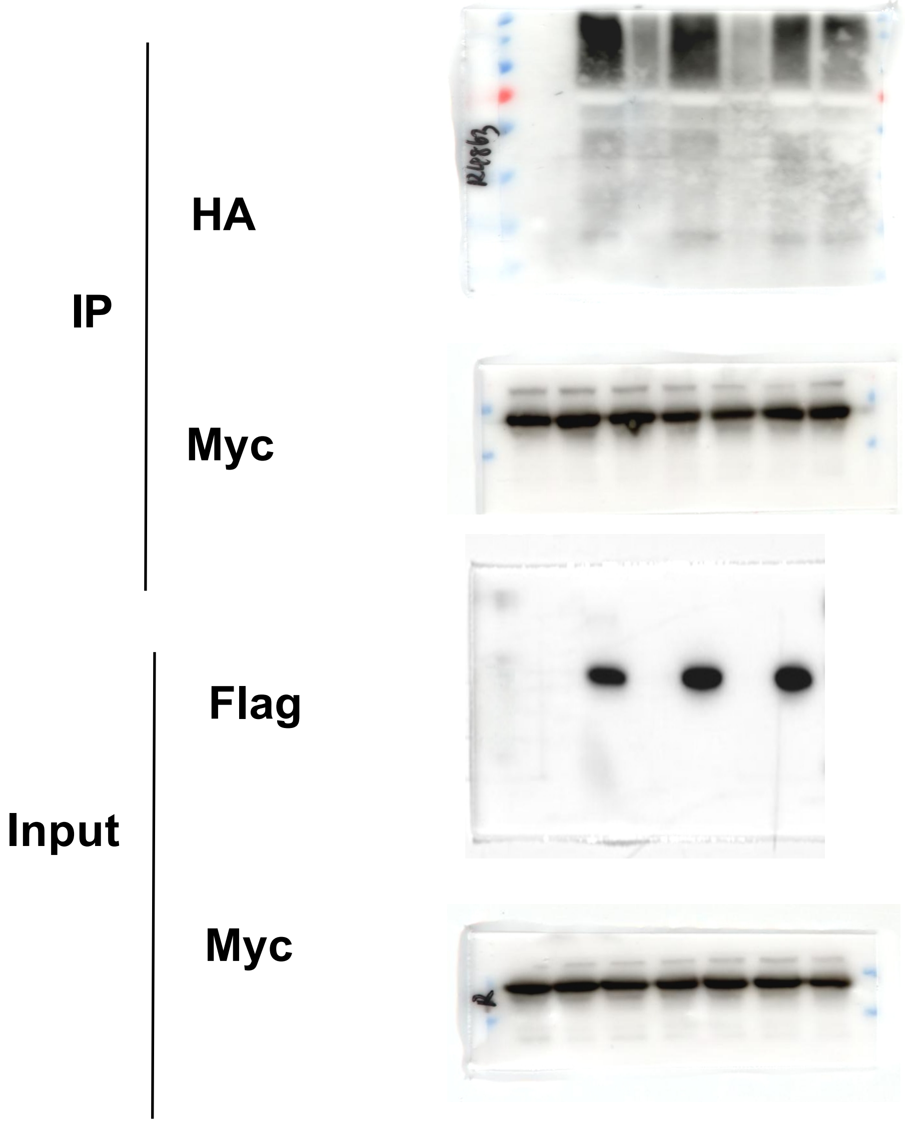

Figure 4

A

IP

Flag

Myc

Input

Flag

Myc

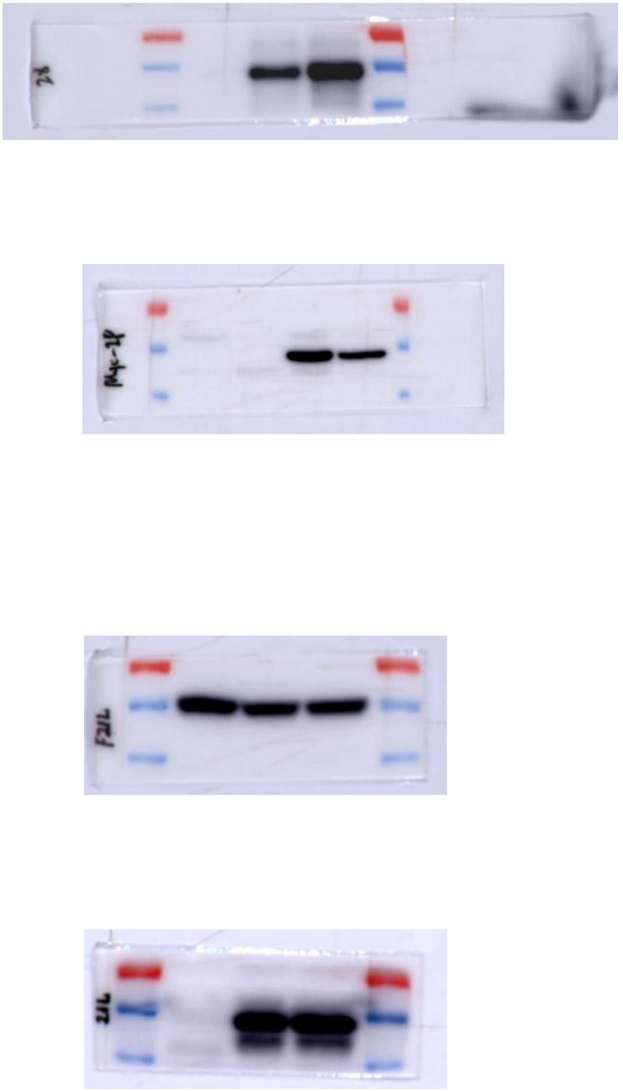

Figure 4

C

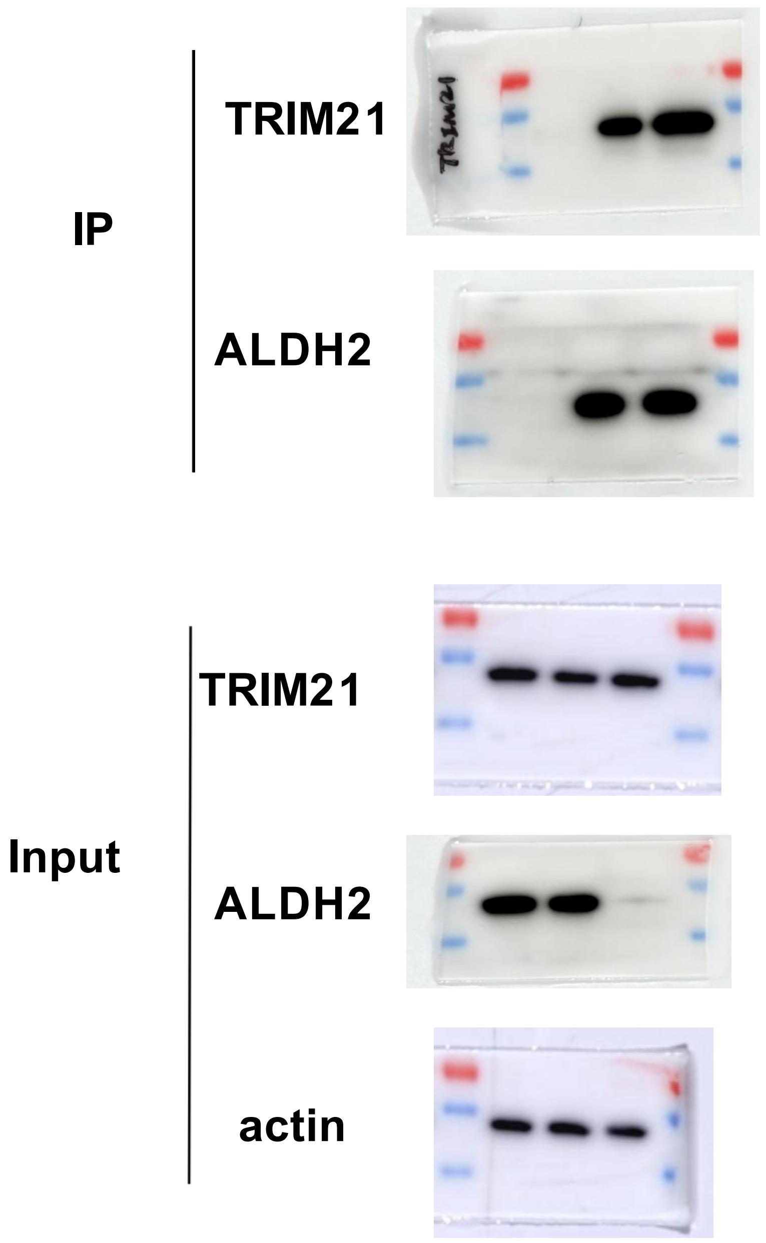

Figure 4

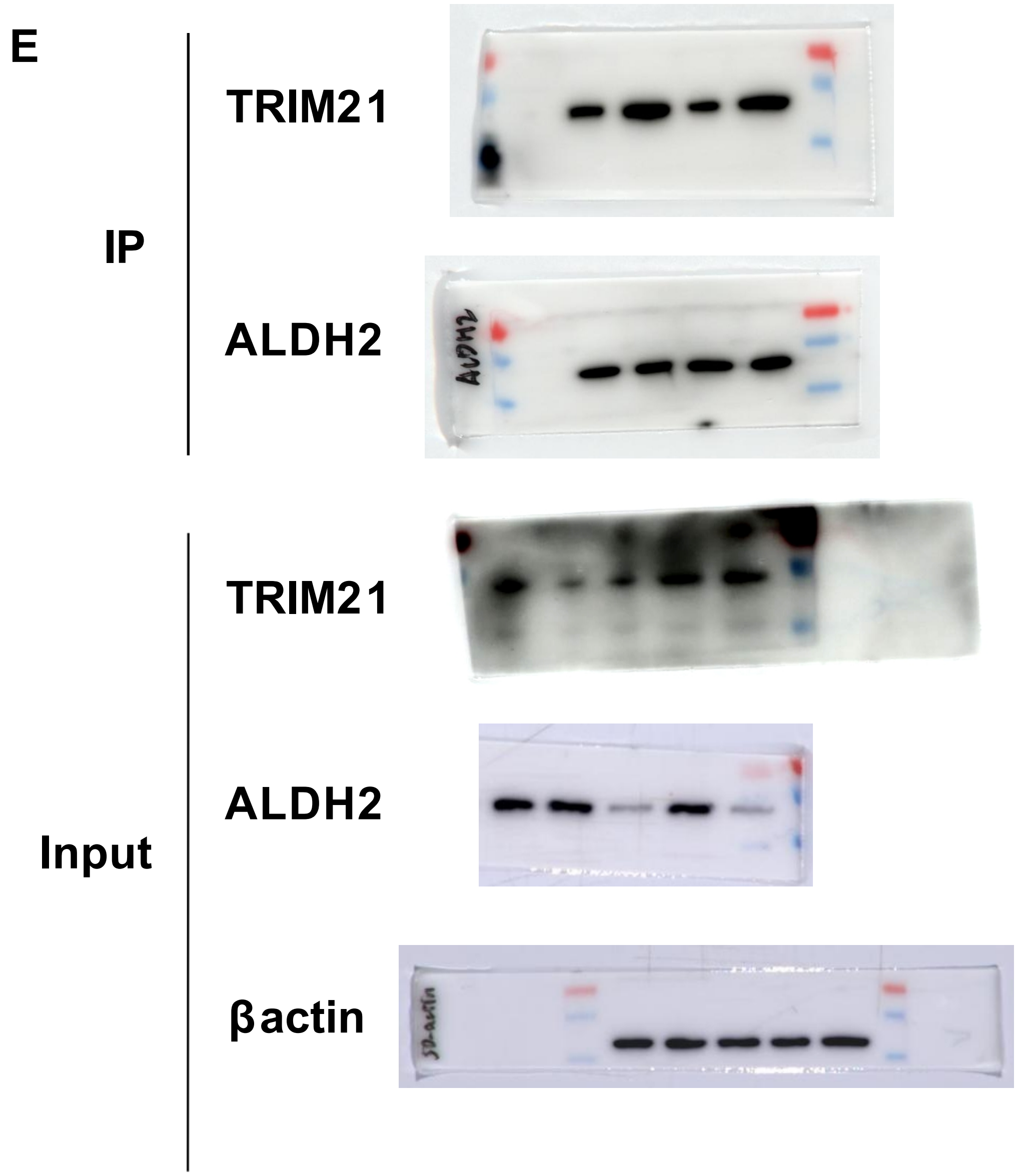

Figure S4

A

TRIM21

βactin

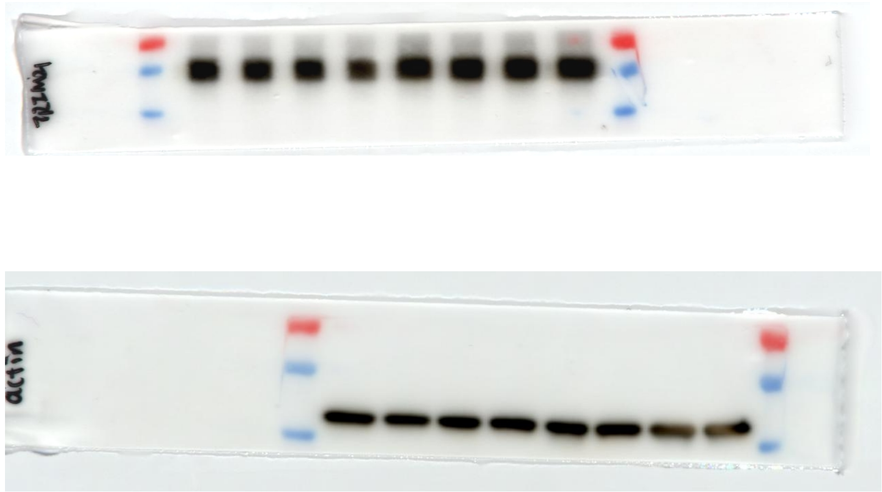

Figure S4

B

ALDH2

βactin

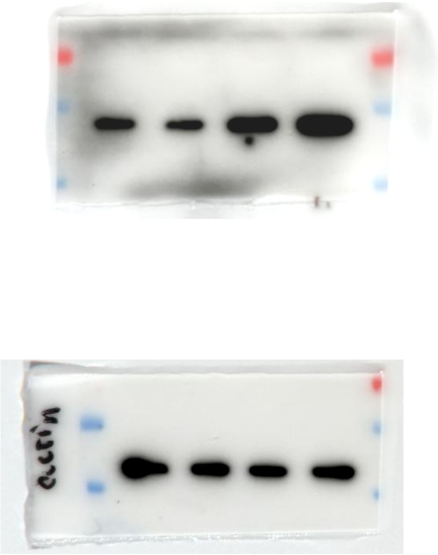

C

TRIM21

ALDH2

βactin

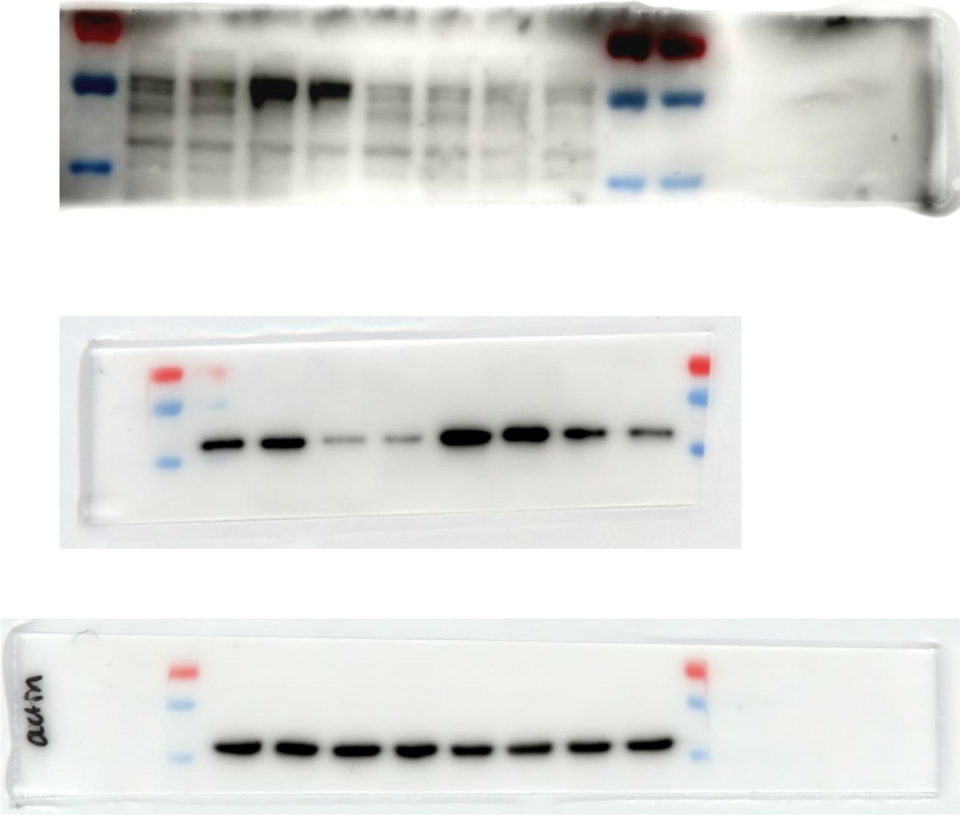

Supplement: Unedited blot and gel images [file jciinsight-11-197555-s151.pdf]
